# Chemical Speciation and Coordination Behavior of 8‑Hydroxyquinoline-2-carboxylic Acid with Divalent Cations in Aqueous Solution: An Irving–Williams Series Study

**DOI:** 10.1021/acsomega.5c06622

**Published:** 2025-11-26

**Authors:** Anna Baryłka, Rafał Bukrym, Izabela Ryza, Clemente Bretti, Sourab Sinha, Rosita Cappai, Gabriele Lando, Oluseun Akintola, Winfried Plass, Beata Godlewska-Zyłkiewicz, Giuseppe Brancato, Demetrio Milea, Sofia Gama

**Affiliations:** † Doctoral School, 49663 University of Bialystok, K. Ciolkowskiego 1K, 15-245 Bialystok, Poland; ‡ Department of Analytical and Inorganic Chemistry, Faculty of Chemistry, University of Bialystok, K. Ciolkowskiego 1K, 15-245 Bialystok, Poland; § Dipartimento di Scienze Chimiche, Biologiche, Farmaceutiche ed Ambientali, CHIBIOFARAM, 18980 Università degli Studi di Messina, Viale F. Stagno d’Alcontres 31, 98166 Messina, Italy; ∥ 19004 Scuola Normale Superiore e CSGI, Piazza dei Cavalieri, 7, 56126 Pisa, Italy; ⊥ Dipartimento di Scienze Chimiche, Fisiche, Matematiche e Naturali, 9312 Università di Sassari, via Vienna 2, Sassari 07100, Italy; # 9378 Institut für Anorganische und Analytische Chemie Friedrich-Schiller-Universität Jena, Humboldtstr. 8, 07743 Jena, Germany; ∇ Istituto Nazionale di Fisica Nucleare (INFN), Largo Pontecorvo, 3, 56127 Pisa, Italy; ○ Centro de Ciências e Tecnologias Nucleares, Instituto Superior Técnico, Universidade de Lisboa, Estrada Nacional 10 (km 139.7), 2695-066 Bobadela LRS, Portugal

## Abstract

In this work, the coordination properties of 8-hydroxyquinoline-2-carboxylic acid (8-HQA, LH_2_) toward Mn^2+^, Fe^2+^, Co^2+^, Ni^2+^, Cu^2+^, and Zn^2+^ are discussed. Stability constants for Mn^2+^, Co^2+^, and Ni^2+^/8-HQA systems were determined by ISE-H^+^ (glass electrode) potentiometry, and those of Cu^2+^ and Zn^2+^/8-HQA by ultraviolet–visible (UV–vis) spectrophotometry, in KCl_(aq)_ at *I* = 0.2 mol dm^–3^ and *T* = 298.2 K. For all systems, three species are formed: MLH^+^, ML, and ML_2_^2–^. 8-HQA proved a good sequestering agent of M^2+^ over a wide pH range, as also shown by the calculated pL_0.5_ values. The stability of the formed metal complexes follows the expected Irving–Williams trend, especially concerning the ML_2_^2–^ species, with log β_120_: 12.45 ± 0.01 (Mn^2+^) < 13.45 (Fe^2+^) < 15.90 ± 0.04 (Co^2+^) < 17.17 ± 0.05 (Ni^2+^) < 20.64 ± 0.03 (Cu^2+^) > 18.78 ± 0.02 (Zn^2+^). This trend is inversely correlated to the M–N bond length determined by quantum mechanical calculations. These studies, together with voltammetry and electron paramagnetic resonance spectroscopy, allowed us to derive information about the coordination modes, structure, and nature of the formed species. Results support the formation of ML_2_^2–^ complexes over possible ML­(OH)^−^, with 8-HQA acting as tridentate in all formed species, including the protonated MLH^+^.

## Graphical abstract

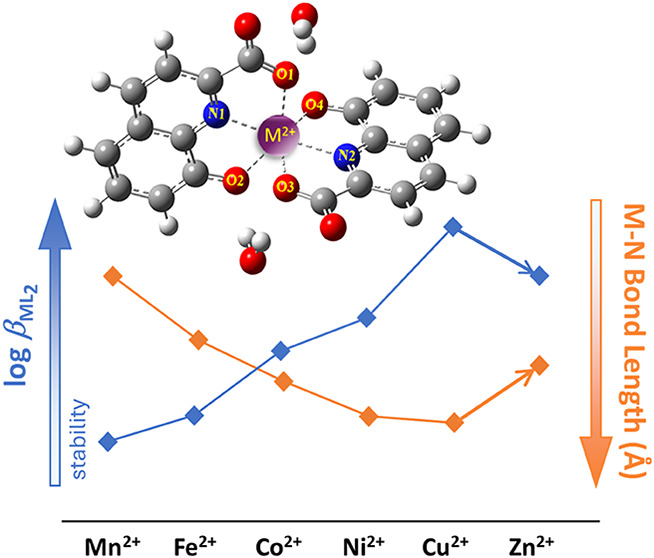

## 1 Introduction

Divalent metal cations such as manganese (Mn^2+^), iron (Fe^2+^), cobalt (Co^2+^), nickel (Ni^2+^), copper (Cu^2+^), and zinc (Zn^2+^), the so-called metals of Irving–Williams series,^1^ are known to be essential in living organisms, playing fundamental roles in several biochemical functions, from enabling enzymes to function, stabilize protein structures, assist in DNA replication, and support cellular respiration and antioxidant defense.^2–5^ Nevertheless, metal cations rarely work in their simple cationic form, and they form complexes with biomolecules that control their reactivity and localization. In general, the balance between functional metal availability and detoxification, the metal homeostasis, is tightly controlled by transporters, chaperones, storage proteins like ferritin, and small-molecule chelators.^4–6^

Metal homeostasis is also fundamental in the balance and quality of microbial community in the intestine, being known that dietary metals have the potential to change the distribution and function of the microbiota, affecting, for example, amino acid metabolism, like that of tryptophan (Trp).^7^ Namely, it is known that zinc deficiency may increase tryptophan depletion and inflammatory signaling, potentially contributing to mood disorders and impaired mucosal immunity.^8^ Furthermore, certain gut microbes that metabolize tryptophan into immunomodulatory indoles also require appropriate levels of metal ions, like iron and manganese, to thrive and perform this function.^9^ Thus, many studies report the association of imbalances in the levels of Trp and its metabolites with a wide range of human pathologies such as depression, schizophrenia, autoimmune disorders, neurodegeneration, and cancer, in particular colorectal cancer (CRC), one of the most malignant ones.^10,11^

From the several metabolites resulting from the kynurenic Trp metabolic pathway, we recently dedicated our work to the study of the chemical speciation of one of its final metabolites, the 8-hydroxyquinoline-2-carboxylic acid (8-HQA, LH_2_), found in high concentrations (0.5–5.0 mmol dm^–3^) in the gut of *Noctuid larvae*, with a remarkable activity as siderophore, regulating the number and diversity of bacteria in the gut microbiome of *Spodoptera littoralis*.^12,13^

This inspired the investigation on the chemical speciation, coordination, and sequestering ability of this ligand toward Fe^2+/3+^, Ga^3+^, and MoO_4_^2–^ ions. Our recent studies demonstrated its ability as a metal chelator, binding iron and gallium ions with remarkable efficiency. We showed that 8-HQA forms stable complexes in environments like the insect gut, suggesting a role in microbial metal scavenging. It could also act as a molybdophore, helping microbes acquire molybdenum for vital enzymes, and, more recently, we found that 8-HQA metal complexes even show antimicrobial activity, making it a potential player in shaping microbial communities.^14–16^

For a larger overview of the chelating ability of 8-HQA, in this work, we present a detailed study on the chemical speciation and coordination properties of 8-HQA toward divalent metal ions of the first transition series in aqueous solution. The binding ability of 8-HQA toward the divalent cations of the so-called Irving–Williams series (i.e., Mn^2+^, Fe^2+15^, Co^2+^, Ni^2+^, Cu^2+^, Zn^2+^) has been investigated in KCl_(aq)_ at *I* = 0.2 mol dm^–3^ and *T* = 298.2 K by ion selective H^+^ glass electrode (ISE-H^+^) potentiometric and ultraviolet–visible (UV–vis) spectrophotometric titrations. Differential pulse anodic stripping voltammetry (DP-ASV) and cyclic voltammetry (CV), EPR (electron paramagnetic resonance spectroscopy), and quantum mechanical calculations have also been performed to derive information about the coordination modes, structure, and nature of formed species.

## 2 Materials and Methods

### 2.1 Chemicals

8-Hydroxyquinoline-2-carboxylic acid (8-HQA, LH_2_) solutions were prepared by weighing analytically pure compounds purchased from Sigma-Aldrich Chemie GmbH (Steinheim, Germany). A minimum known amount of ethanol (POCH S.A., Gliwice, Poland) was used to promote initial ligand solubilization in water, never exceeding, in any case, 2% v/v. Metal ions aqueous solutions were prepared by weighing the corresponding chloride salts (i.e., MnCl_2_·4H_2_O, CoCl_2_·4H_2_O, NiCl_2_·6H_2_O, CuCl_2_·2H_2_O, ZnCl_2_), purchased from Sigma-Aldrich Chemie GmbH, Steinheim, Germany, of analytical grade purity, and were standardized against EDTA (POCH S.A., Gliwice, Poland) standard solutions.^17^ KCl (Merck KGaA, Darmstadt, Germany) aqueous solutions were prepared by weighing the pure salt, previously dried at *T* = 383.2 K for at least 2 h. KOH and HCl solutions were prepared by diluting the concentrated ampules (POCH S.A., Gliwice, Poland) and were standardized against potassium hydrogen phthalate (Standard Reference Material, Merk, Germany) and tris­(hydroxymethyl)­aminomethane (Sigma-Aldrich Chemie GmbH, Steinheim, Germany), respectively, previously dried for at least 2 h (the former at *T* = 383.2 K, the latter at *T* = 353.2 K). Solutions were prepared in ultrapure water (*R* = 18 MΩ cm^–1^) and grade A glassware.

### 2.2 ISE-H^+^ Potentiometry

An Orion Star T900 Series Potentiometric Titrator (Thermo Scientific, Waltham, MA, USA) equipped with an automatic buret and a combined ISE-H^+^ glass electrode Orion 8102BNUWP ROSS Ultra (Thermo Scientific) was used for potentiometric titrations (estimated accuracy was 0.2 mV and 0.002 cm^3^ for electrode potential and titrant volume readings, respectively). All titrations were carried out at *T* = 298.2 ± 0.1 K in a thermostated glass cell under magnetic stirring and bubbling argon through the solution to exclude/prevent O_2(g)_ and CO_2(g)_ dissolution. The titrand solution consisted of various amounts of 8-HQA (0.9 ≤ *c*_L_/mmol dm^–3^ ≤ 1.2), metal ion (0.3 ≤ *c*_M_/mmol dm^–3^ ≤ 1.2), a slight excess of HCl_(aq)_ (8.0 ≤ *c*_H_/mmol dm^–3^ ≤ 10.0), and KCl_(aq)_ in order to reach the pre-established ionic strength value of *I* = 0.2 mol dm^–3^. To study the metal–ligand interactions, all potentiometric measurements were performed considering different metal/ligand ratios (1:1 ≤ *c*_M_/*c*_L_ ≤ 1:3). 25 cm^3^ of the titrand solutions were titrated with standardized KOH_(aq)_ up to pH ∼11–12 or until the formation of sparingly soluble species. For each titration, the total number of experimental points collected ranged between 80 and 100. Before each measurement, the electrode was calibrated in terms of free hydrogen ion concentration [H^+^] (not activity) using independent titrations of HCl_(aq)_ with standardized KOH_(aq),_ in the same experimental conditions (temperature, ionic medium, and ionic strength) as the systems under study.^18^

### 2.3 UV–vis Spectrophotometry

A Thermo Scientific Evolution One Plus UV–vis spectrophotometer (Thermo Scientific, Waltham, MA, USA) was used to perform UV–vis spectrophotometric measurements. Spectra were recorded in the wavelength range of 200 ≤ λ/nm ≤ 500, using a VERSA optical fiber probe, with a fixed 1 cm path length. All measurements were performed by titrating 30 cm^3^ of the titrand solution with standardized KOH_(aq)_ up to pH ∼ 10.5, in the same experimental conditions of temperature and ionic strength as the potentiometric titrations (*T* = 298.2 ± 0.1 K, *I* = 0.2 mol dm^–3^ in KCl_(aq)_). Various metal to ligand ratios were used, i.e., 1:1 ≤ *c*_M_/*c*_L_ ≤ 1:3 and different concentrations (5 × 10^–6^ ≤ *c*_L_ ≤ 5 × 10^–5^ mol dm^–3^). The pH of each solution was measured using the potentiometric system described above.

### 2.4 Voltammetry

DP-ASV and CV experiments were carried out for the Mn^2+^ /8-HQA system at *T* = 298.2 ± 0.1 K and *I* = 0.2 mol dm^–3^ in KCl_(aq)_ in thermostated cells using a Metrohm 663 VA Stand (Series 05) workstation, equipped with a three-electrode system supplied by Metrohm, consisting of (i) a multimode mercury electrode (MME, model 6.1246.020) filled with 99.9999% mercury (electronic grade, from Aldrich) working in SMDE mode (Static Mercury Drop Electrode); (ii) a glassy carbon (GC) auxiliary electrode (AE) (model 6.1247.000); (iii) a double junction Ag/AgCl/KCl (3.0 mol dm^–3^) reference electrode (RE) (model 6.0728.000 + 6.1245.000). The workstation was connected to a μAutolab type III potentiostat/galvanostat (Eco Chemie) with an IME663 interface (Eco Chemie). The whole system was controlled by the GPES v. 4.9 software (Eco Chemie). The free hydrogen ion concentration [H^+^] in the experiments was measured before and after each voltammetric run, using the same apparatus and procedure. For Mn^2+^, it is not possible to work in very acidic solutions due to the presence of H^+^ reduction in the same electrochemical window.^19^ As such, the following experimental conditions were adopted for DP-ASV experiments: purge time of 300 s, deposition of 300 s at −1.7 V, equilibration of 10 s at −1.7 V without stirring, scan between −1.7 and −1.3 V with a step potential of 2 mV every 0.1 s (scan rate 20 mV s^–1^), modulation time of 0.05 s, and modulation amplitude of 0.1 V. For CV measurements, the scan was the result of the average of ten replicates in the same electrochemical window, taken without accumulation time at various scan rates in the range of 50–500 mV s^–1^; the best results were reported for a scan rate of 100 V s^–1^. Under these experimental conditions, it was possible to obtain resolved peaks in the pH range between ∼4 and ∼10. In the same experimental conditions, 8-HQA did not show any electrochemical process.

Two kinds of DP-ASV titrations were performed: the first, aimed at determining the number of ligands bound to Mn^2+^, was carried out by adding 8-HQA to Mn^2+^ solutions (4 × 10^–6^ ≤ *c*_Mn_/mol dm^–3^ ≤ 5 × 10^–6^; linearity tests were performed in the range of 1 × 10^–6^ ≤ *c*_Mn_/mol dm^–3^ ≤ 1 × 10^–4^ mol dm^–3^) at pH ∼ 9.5 up to *c*_L_ ∼ 2 × 10^–4^ mol dm^–3^; the second consisted of acid–base titrations, in the range of 3.7 ≤ pH ≤ 10.9, of solutions containing both Mn^2+^ (at the same above-reported concentrations) and 8-HQA, at various fixed *c*_L_/*c*_M_ ratios (5 ≤ *c*_L_/*c*_M_ ≤ 15), to evaluate eventual changes in the number of protons and ligands of complex­(es) along with pH variation.

More information on experimental details can be found in a dedicated section in Supporting Information and in refs 20,21

### 2.5 Electron Paramagnetic Resonance Spectroscopy

The continuous-wave (CW) EPR spectra for the frozen solutions at liquid nitrogen temperatures were recorded by using an X-Band EPR-ELEXSYS E580 Spectrometer (Bruker BioSpin, Germany) equipped with an SHQE resonator (Bruker ER4122SHQE) at a microwave frequency of 9.33 GHz. Low temperatures were achieved with a Quartz Finger Dewar insert coupled to a digital temperature control system ER4121VT from Bruker. Measurements were carried out using 4 mm EPR Quartz tubes. All spectra were acquired using a microwave power of 1.5–4.7 mW, with a modulation amplitude of 0.3 or 0.4 mT at *T* ∼ 100 K. Simulations of the obtained spectra were carried out using EasySpin^22^ within the Matlab environment.

### 2.6 Quantum Mechanical Calculations

Molecular structures of the various metal complexes were obtained by geometry optimization using density functional theory (DFT) with the Minnesota functional M06 ^23^ and the 6–311++G­(d,p) basis set. The molecular structures were optimized with different spin multiplicities to identify the minimum-energy spin configurations for each metal complex. The ground-state minimum-energy geometry was verified in each case by using a harmonic frequency analysis. Based on experimental findings, the metal cations were considered in their corresponding high-spin state. All calculations were performed using Gaussian16 ^24^ software. In order to study the solvent effect on the chemical species, the Self-Consistent Reaction Field (SCRF) approach was used with the conductor-like polarizable continuum model (CPCM)^25,26^ at a dielectric constant, ε = 78, to mimic the effect of water as implemented in Gaussian16.

### 2.7 Calculations on Chemical Speciation

Suitable computer programs were used for processing data obtained by various analytical techniques. The BSTAC software,^27^ which minimizes the sum of square errors in electrode potential readings, was used to handle potentiometric data, refining the stability constants of formed species in different M^2+^/8-HQA systems and all parameters of the potentiometric titrations (standard formal potential (*E* ^0^), ionic product of water (*pK*_w_), and the acidic junction coefficient (*j*_a_)). The UV–vis spectrophotometric data were analyzed by the HypSpec2014 program,^28^ which allowed the determination of the stability constants and the molar absorbance spectra of each absorbing species. PyES program^29^ was used to draw the speciation diagrams and to calculate species formation percentages.

The stability constants reported in this work, including ligand protonation (*p* = 0) and cations hydrolysis (*q* = 0 and *r* < 0), are expressed considering the overall equilibrium described in eq 1, where L stands for the fully deprotonated 8-HQA and M = Mn, Fe, Co, Ni, Cu, or Zn

1 $$pM^{2+}+qL^{2-}+rH^{+}⇄M_{p}{L_{q}H_{r}}^{\left(2p-2q+r\right)}\log\beta_{\mathrm{pqr}}$$

Though this is not strictly correct, to better identify various species, those with *p* = 1 or *q* = 1 are denoted throughout the text as mononuclear and monoligated, respectively, while those with *p* > 1 or *q* > 1 are designated as polynuclear and polyligated.

When not relevant and for simplicity, the charges of the various species are omitted.

## 3 Results and Discussion

### 3.1 Stability Constants and Speciation Model

For an accurate assessment of the chemical speciation of a given M^2+^/8-HQA system, knowledge of the acid–base properties of both the metal cations (hydrolysis) and the ligand (protonation), under the same experimental conditions, is fundamental. Protonation constants of 8-HQA, at *T* = 298.2 ± 0.1 K and *I* = 0.2 mol dm^–3^ in KCl_(aq)_, were already determined in our previous work^15^ and are reported as Supporting Information (Table S1), following the protonation sequence represented in Scheme 1. For the studied metal cations, their hydrolysis constants were taken from literature^30,31^ and, if necessary, recalculated for our experimental conditions using common models for the dependence of stability constants (and activity coefficients) on medium, ionic strength, and temperature (see, e.g., refs in^16,29,32^ ), and are reported in Table S2.

**Scheme 1:**
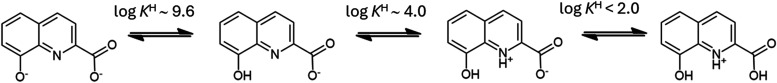
Protonation Sequence of 8-HQA

All M^2+^/8-HQA systems were initially studied by potentiometric titrations at various metal to ligand ratios (1:1 ≤ *c*_M_/*c*_L_ ≤ 1:3) to investigate the possible formation of M*_p_* L*_q_* H*_r_* species with *q* > 1 (observed for other similar systems^15,16,33^ ). However, the formation of sparingly soluble species in the case of Cu^2+^ and Zn^2+^ systems in the conditions of potentiometric experiments restricted the investigable pH and concentration ranges, hampering the collection of a suitable amount of experimental data for an accurate determination of the stability constants of M*_p_* L*_q_* H*_r_* species of these two cations. This fact limited the potentiometric results to Mn^2+^, Co^2+^, and Ni^2+^ only (data for Fe^2+^ are already reported in ref^15^). As suggested in these cases,^34^ both Cu^2+^/8-HQA and Zn^2+^/8-HQA systems were thus studied by UV/vis spectrophotometry, which allowed us to work at lower concentrations, extending the investigated pH range up to pH ∼ 10.5, without precipitation.

Importantly, this approach requires the knowledge of molar absorbance spectra of 8-HQA and its protonated species (Figure S1, reported in our previous work^14^), to include them as input in the HypSpec2014 software^28^ for the determination of stability constants from UV–vis data. An example of UV–vis titration of Cu^2+^/8-HQA system (*c*_M_/*c*_L_ = 1:2) in the 220 ≤ λ/nm ≤ 500 wavelength range is reported in Figure 1a, together with the calculated molar absorptivity spectra of each species (see Figure 1b) obtained after the experimental analysis. The analogue spectra for the Zn^2+^/8-HQA system are reported as Supporting Information (Figure S2).

**Figure 1:**
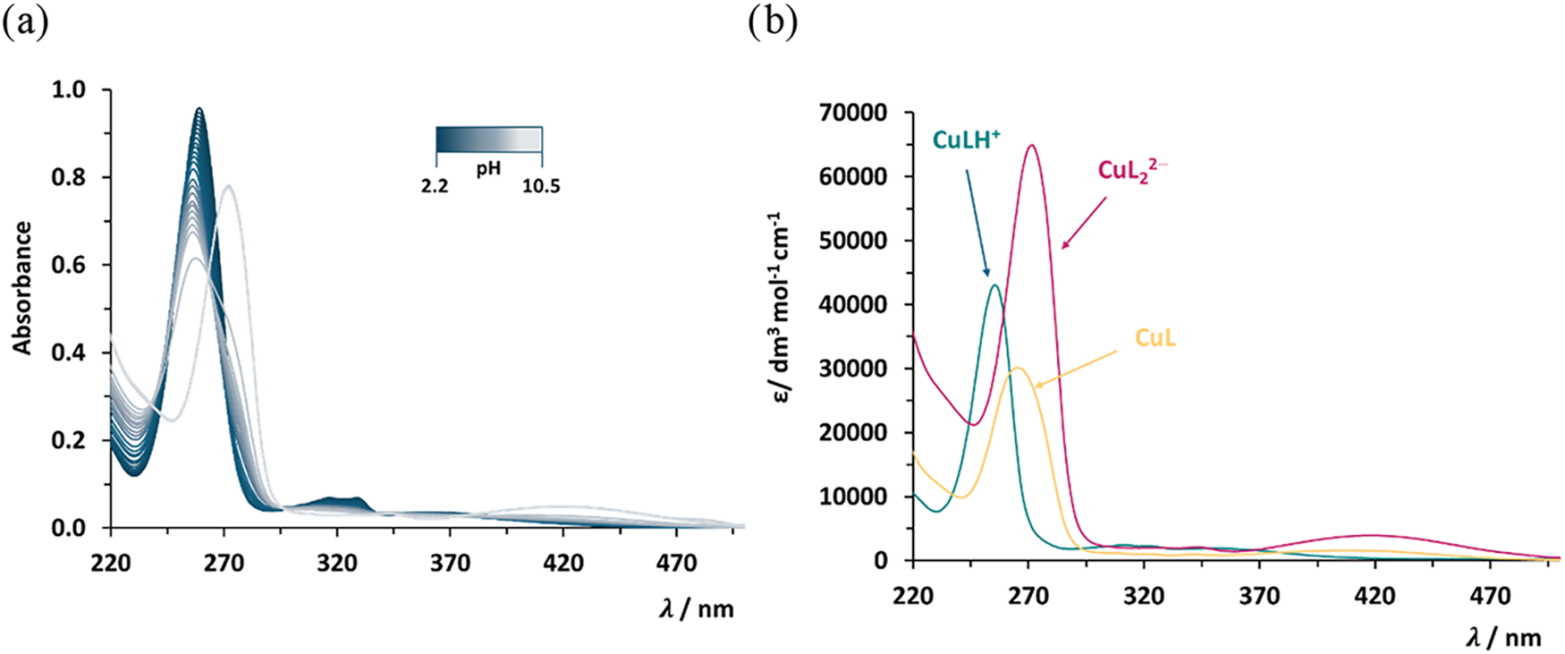
(a) Experimental UV–vis spectra of Cu^2+^/8-HQA system vs pH (*c*_L_ = 2 × *c*_M_ = 2.57 × 10^–5^ mol dm^–3^, in *I* = 0.2 mol dm^–3^ KCl_(aq)_ and *T* = 298.2 K); (b) calculated molar absorptivity spectra of CuL, CuLH^+^, and CuL_2_^2–^ species.

For all studied systems, experimental data analysis gave evidence of the formation of only three species, namely, ML, MLH^+^, and ML_2_^2–^. The corresponding stability constants are listed in Table 1.

**Table 1:** Experimental Stability Constants of the M*_p_* L*_q_* H*_r_* Species Determined at *T* = 298.2 ± 0.1 K and *I* = 0.2 mol dm^–3^ in KCl_(aq)_

species	*p*:*q*:*r*	log β*_pqr_* ^a^					
		Mn^2+^	Fe^2+^ ^b^	Co^2+^	Ni^2+^	Cu^2+^	Zn^2+^
MLH^+^	1:1:1	12.66 ± 0.01	13.60	12.44 ± 0.03	12.68 ± 0.01	15.82 ± 0.01	15.28 ± 0.01
ML	1:1:0	6.84 ± 0.01	9.62	9.20 ± 0.01	10.31 ± 0.01	12.05 ± 0.01	9.29 ± 0.05
ML_2_^2–^	1:2:0	12.45 ± 0.01	13.45	15.90 ± 0.04	17.17 ± 0.05	20.64 ± 0.03	18.78 ± 0.02

a log β*_pqr_* refer to equilibrium: p M + q L + r H = M*_p_* L*_q_* H*_r_*, ±95% confidence interval.

b From ref^15^.

### 3.2 Reliability of the Speciation Model and the Coordination Behavior of 8-HQA: Critical Aspects

The main concerns about metal complexes of 8-HQA regard the nature of species formed in solution (MLH*_r_* vs ML*_q_* H*_r_* species with *q* > 1, especially ML_2_H*_r_*), as well as their binding mode (bidentate vs tridentate and, in the former case, through carboxylic or phenolic oxygen). Both aspects are critical to understand the behavior of 8-HQA in the presence of various metal cations in aqueous solution.

#### 3.2.1 Reliability of the Speciation Model: Mono- vs Bis-Ligated Species

According to literature findings, 8-HQA may form mono- and/or bis-ligated complexes and even tris-ligated in some particular conditions.^14–16,33^ The preference toward one or the other kind of species is dependent on the nature of the complexed metal ion but mainly on the conditions of various studies (e.g., the metal to ligand ratio, pH, concentrations of reagents). However, it is crucial to stigmatize here that pre-established conditions are, nevertheless, not sufficient to guarantee the formation of only one kind of species. In fact, in our previous studies,^15,16^ we observed the formation of both mono- and bis-ligated 8-HQA complexes of Fe^3+^ and Ga^3+^ independently of the metal to ligand ratio (*c*_M_/*c*_L_ = 1:1 or 1:2), while only monoligated species were observed with MoO_4_ ^2–14^ up to a metal-to-ligand ratio of *c*_M_/*c*_L_ = 1:3. Reversely, only bis-ligated metal complexes were synthesized and isolated in the solid state by McDonald et al.,^33^ despite the use of a *c*_M_/*c*_L_ = 1:1 ratio. As also stated by the same authors, “there is no necessary correspondence between solution species and species isolated in the solid state.” In fact, in the same conditions, but in solution, they only observed the formation of various monoligated species for several M^m+^/8-HQA systems. As such, a proper experimental design (e.g., planning experiments at various analytical concentrations and ratios and/or adopting a multitechnique approach) is crucial to obtain suitable results for the definition of the speciation model of a given system, distinguishing and/or identifying various species that may exist.

This may help, for example, to distinguish between the possible formation of mixed hydroxide complexes (e.g., ML­(OH)^−^) and/or bis-ligated species (e.g., ML_2_^2–^). In fact, if the ML­(OH)^−^ species occurs at the same pH range in which the ligand is still protonated (as may be the case for 8-HQA), it can be proven (e.g., by solving mass balance equations) that, when working only at a fixed *c*_M_/*c*_L_ ratio (in particular at *c*_M_/*c*_L_ = 1:1 or when the two concentrations are relatively low and/or not so different), the amount of proton displaced from one water molecule coordinated to the metal center of the ML species to form ML­(OH)^−^ is comparable to that occurring when a second (protonated) ligand binds to ML to form ML_2_^2–^, so that one process almost mimics the other, making the inclusion in the speciation model of one or another species almost equivalent. As such, ML_2_^2–^ and ML­(OH)^−^ are not easily distinguishable, both resulting in fitting processes that often give analogous results (in terms of quality) when considering one species or the other in the model. That is why it is fundamental to perform experiments at different analytical concentrations and ratios, combining potentiometric and/or spectrophotometric alkalimetric/acidimetric titrations with other techniques that make it possible to distinguish between different possible species, evaluating, for example, their stoichiometry (see, e.g., discussion in the section dedicated to voltametric studies).

## 3.2.2 Coordination Behavior: Tri- vs Bidentate and Coordinating Groups

8-HQA is a rigid, highly preorganized ligand^33^ able to form up to two 5-membered chelate rings when it coordinates metal ions through its three functional groups (i.e., the quinolinic nitrogen, the phenolate in position 8, and the carboxylate in position 2), acting as tridentate (Scheme 2a). However, beside the “central” nitrogen, one of the two “side” O donor groups may be less or not involved in metal coordination, making 8-HQA act as bidentate (thus forming only one chelate ring, Scheme 2b,c). Furthermore, one or both among the carboxylate and the phenolate may coordinate the metal ion even if they are protonated, leading to the possible formation of MLH^+^ and/or MLH_2_^2+^ species, though the proton on the carboxylate moiety is very acidic (log *K* ^H^ < 2). Finally, the ligand preorganization, resulting from the rigidity imposed by the quinoline ring and the position of the two side groups, is particularly suitable to favor the formation of octahedral bis-ligated (or even tris-ligated, if acting as a bidentate ligand)^15^ metal complexes.^14–16,33^

**Scheme 2:**
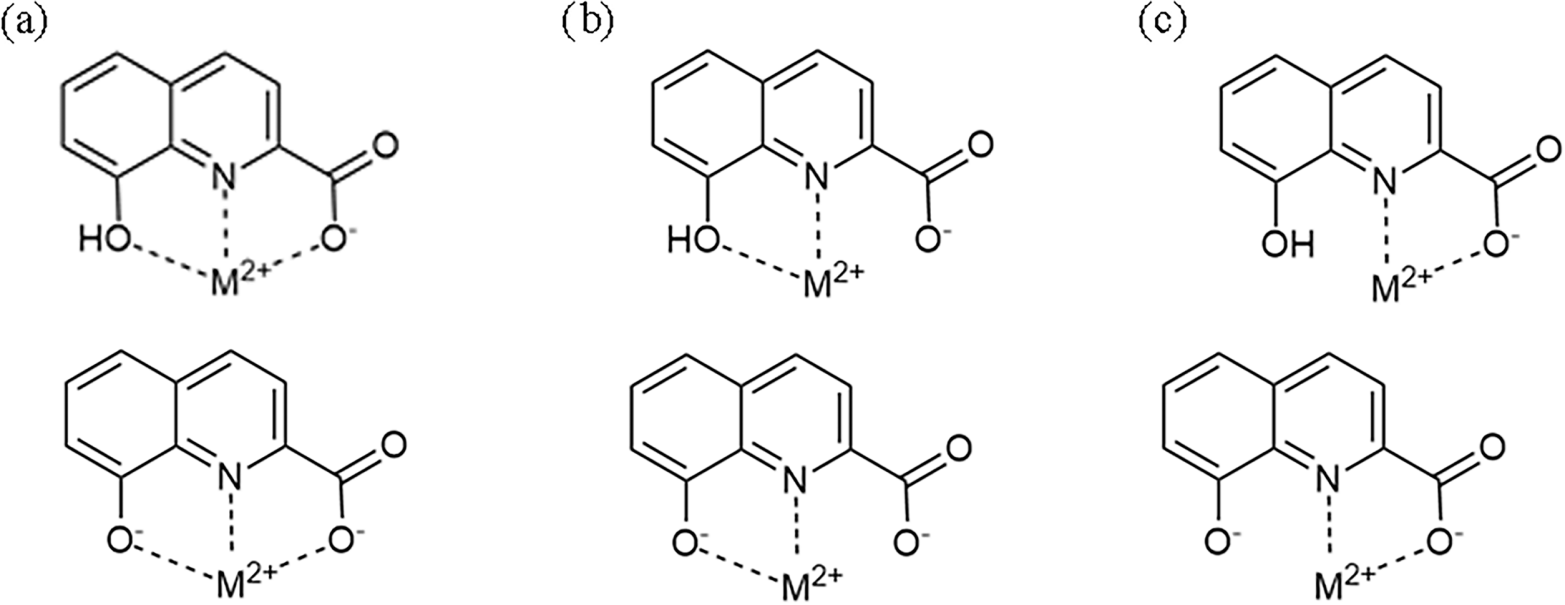
Possible Binding Modes of 8-HQA Metal Complexes Where L Coordinates in a (a) Tridentate Mode with Either Protonated or Deprotonated Phenolate; (b) Bidentate via Phenolic/Phenolate Group; (c) Bidentate via Carboxylate

Altogether, this plethora of possible binding modes affects not only the structure of formed complexes but also their nature, their stability in solution, and potential activity (e.g., in biology and medicine).

As such, to get further insights on the nature of species formed (and thus to confirm the proposed speciation models), as well as on the coordination behavior of 8-HQA toward the cations investigated in this article, we performed further studies on some selected M^2+^/8-HQA systems by other techniques than those used to determine the stability constants of its metal complexes in aqueous solution. As such, we planned voltammetric measurements on the Mn^2+^/8-HQA system to verify, mainly, the formation of other species than MLH*_r_*; EPR studies on Mn^2+^/8-HQA and Co^2+^/8-HQA to get insights on the spin states and the coordination of the ligand(s) to the metal center; quantum mechanical calculations to further support experimental results. Results for each technique are discussed separately in the following sections.

### 3.3 Voltammetric Study on the Mn^*2+*^/8-HQA System

Several voltammetric experiments were performed on the Mn^2+^/8-HQA system to give evidence about the number of ligands that can be coordinated to the metal center, i.e., on the nature and/or stability of complexes formed by the target metal ion and a ligand of interest, including protonated and/or hydroxo-species (see, e.g., refs in^34^ and in chapters 1 ^35^ and 2 ^36^ of ref^37^).

The analysis of the DP-ASV peaks recorded during the acid–base titrations performed at a fixed *c*_L_/*c*_M_ ratio (*c*_L_/*c*_M_ = 14.2, *c*_M_ = 4 × 10^–5^ mol dm^–3^) shows the behavior of a fully labile system for pH < 4.2 (Figure 2).

**Figure 2:**
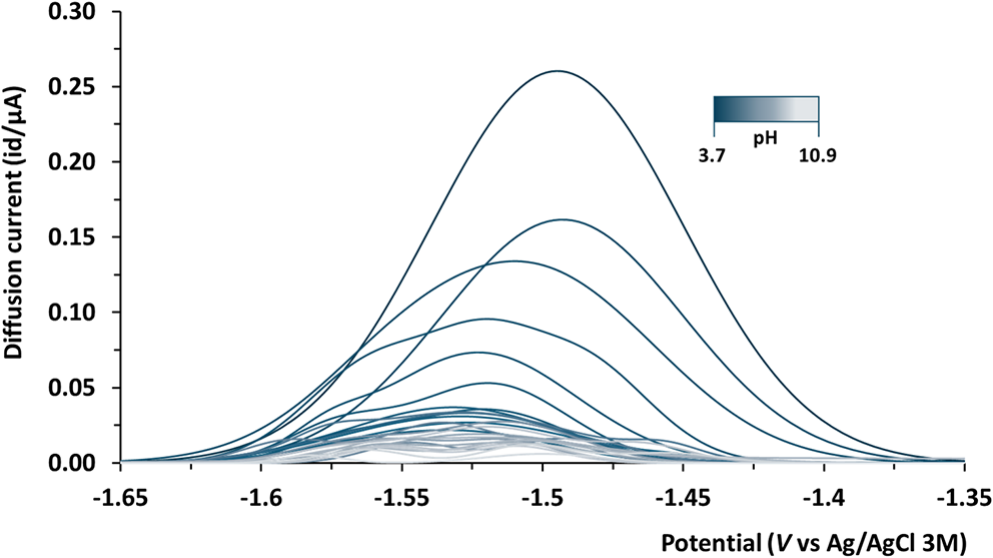
Voltammograms recorded during a voltametric titration at a fixed ratio of *c*_L_/*c*_M_ = 14.2 (*c*_M_ = 4 × 10^–5^ mol dm^–3^) as a function of pH in the range of 3.69 – 10.92 at *T* = 298.2 K and at *I* = 0.2 mol dm^–3^ in KCl_(aq)._.

At pH > 4.2, this condition is lost, as observed by an exponential decrease of the peak height, and the formation of a shoulder (centered at more negative potential values) on the metal reduction peak, which splits from the parent peak only at pH > 9.5. This behavior is typical of the coexistence of labile and nonlabile species, in which the determination of the equilibrium constants is a challenging task. Besides, according to literature, this can be seen as a first indication of the formation of polyligated species such as ML_q_.^38,39^ In the same direction, though the presence of nonlabile species hampers the exact determination of the stoichiometry of formed complexes from the classical Δ*E* (i.e., the shift of the deposition potential of the free metal ion, *E*_parent_, due to complex formation, in which Δ*E = E*_parent_ *– E*_labile_) vs. pH (acid–base titration) or vs −log *c*_L_ (ligand titration) plots (see Supporting Information), the observed behavior supports the formation of bis-ligated rather than hydrolytic species, being coherent with the proposed speciation model for the Mn^2+^/HQA system. In fact, in the Δ*E* vs. pH plots (see, e.g., Figure S5), despite the scattered nature of reported currents (due to the low intensities), three regions can be roughly identified, in which the slope changes at pH ∼ 4.2 and pH ∼ 9.5. The slope observed in the first region (i.e., 56 mV per pH unit) is consistent with the formation of a protonated species (e.g., MLH^+^). In the second region, the slightly positive slope (i.e., 2 mV per pH unit) indicates that the complexation between metal cation and the ligand is not associated with a proton exchange, suggesting the presence of a “less protonated” (e.g., ML) species. The change of the slope at pH ∼ 9.5 is ∼7 mV, which is really not significant for the conditions of experiments and discourages the hypothesis of the formation of a more deprotonated complex (e.g., from ML to ML­(OH)^−^). Conversely, at pH > 9.5, the Δ*E* vs −log *c*_L_ (ligand titration) plots (e.g., Figure S6) exhibit a clear and significant difference in slope with increasing ligand concentration, strongly indicating a variation in the number of ligands bound to Mn^2+^, i.e., at least two distinct ML*_q_* complexes are formed (like, e.g., ML and ML_2_^2–^).

### 3.4 EPR Study on Mn^*2+*^/8-HQA and Co^*2+*^/8-HQA Systems

Continuous-wave electron paramagnetic resonance spectroscopy (CW-EPR) was used to conclusively determine the spin states of Mn^2+^ and Co^2+^. The EPR spectra of both ions mixed with 8-HQA in the appropriate stoichiometries and at varying pH values were recorded as their aqueous solutions at *T* ∼ 90 K. In addition, spectra of aqueous solutions of the metal ions without the ligand were recorded to provide a benchmark spectrum for comparison.

The spectra for the Mn­(II) systems are displayed in Figure 3. In the EPR spectra of Mn^2+^ without 8-HQA, the expected six lines arising from ^55^ Mn­(II) (*I* = 5/2) can be seen.^40^ Simulation of the spectrum led to the following parameters: *g*_eff_ = 2.00, *A*_iso_ = 9.5 mT. This is consistent with a high-spin Mn­(II) center within an octahedral geometry.^41^ The addition of 8-HQA in a *c*_M_/*c*_L_ = 1:1 ratio resulted in a similar spectrum, but with the appearance of an additional transition at low fields (about 130 to 195 mT), corresponding to a *g*-value of ∼4.4. This new band would become more prominent with a rise in the pH to 6.3 and then 8.5. Furthermore, at basic pH, there was an increased level of underlying broadening in the spectra in addition to the initial six lines from the Mn­(II) center. Mixtures with *c*_M/_ *c*_L_ = 1:2 ratios showed a similar behavior at pH = 3.6, with the spectrum mostly dominated by the Mn^2+^ aquo species. However, at pH = 6.3 and 8.1, an enhanced broadening was observed, much more prominent than in the case of a *c*_M_ */c*_L_ = 1:1 ratio. In both cases, this broadening at higher pH is likely due to the formation of MnL_n_ complexes (where *n* = 1 or 2). This broad shape has been found in Mn­(II) within ligand fields having low symmetry.^42^ In such cases, the Mn­(II) ions are known to give rise to “powder-type” EPR spectra, especially when measured at microwave frequencies around the X-band.^43^ It has been suggested that this arises from inhomogeneous broadening and possible strain in the zero-field splitting. In addition, the band at *g* = 4.4 has been suggested to result from the transition between the third and fourth Kramers’ doublets of Mn­(II).^44,45^

**Figure 3:**
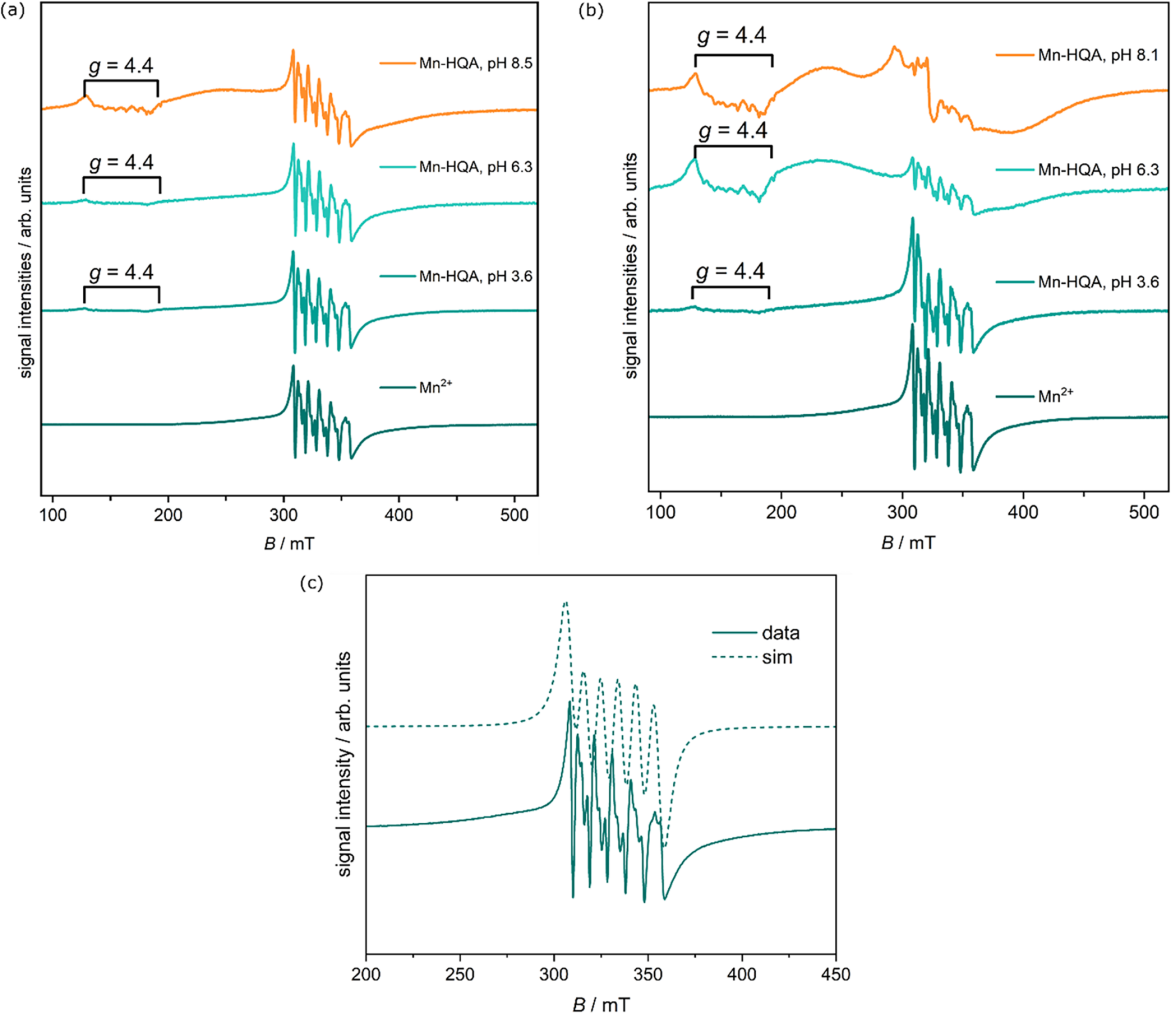
(a) EPR spectra of Mn^2+^/8-HQA system at *T* ∼ 90 K. (a) *c*_M_/*c*_L_ = 1:1 (*c*_M_ = *c*_L_ = 1.2 mmol dm^–3^), pH = 3.6, 6.3, and 8.5; (b) *c*_M_/*c*_L_ = 1:2 (*c*_M_ = 0.6 mmol dm^–3^, *c*_L_ = 1.2 mmol dm^–3^), pH = 3.6, 6.3, and 8.1; (c) experimental (solid line) and simulated (dotted line) EPR spectrum of Mn^2+^ aquo complex (without 8-HQA), pH ∼ 2.

EPR spectra were also acquired for the Co^2+^ complexes, and no spectrum could be observed in any of them (see Figure S7). This is expected for high-spin Co­(II) and is due to the rapid spin–lattice relaxation, which would have the effect of broadening the lines at relatively higher temperatures.^46^ On the other hand, a low-spin Co­(II) would display a signal centered at around *g* = 2.0, which would then be split into eight lines arising from the hyperfine coupling to the ^59^Co nucleus (*I* = 7/2).^47^

Overall, both Mn^2+^ and Co^2+^ can be clearly established to exist in their high-spin state. This information was fundamental to proceed with quantum mechanical calculations.

### 3.5 Quantum Mechanical Calculations

DFT calculations were performed on the M^2+^/8-HQA complexes to obtain some insights into the binding mode, geometry, and energetic stability of the chemical species previously identified. Notably, most of the following considerations can also be derived from a thorough comparison of the ligand protonation (Table S1) and the stability constants of its metal complexes (Table 1). However, these calculations surely give complementary information with respect to the above-mentioned comparison.

In the cases of Mn^2+^ and Co^2+^, a more detailed study was carried out. Various scenarios have been taken into consideration. For example, we investigated whether the ligand binds to the metal as either a bidentate or a tridentate chelating agent and how the monoprotonated 8-HQA binds the metal ion in the MLH^+^ species. Since both Mn^2+^ and Co^2+^ in their high-spin state are predominantly octahedral, coordination was completed by water molecules when necessary.

#### 3.5.1 ML_2_ Species

The optimized structure of MnL_2_^2–^ species is reported in Figure 4a and shows how 8-HQA preferentially acts as tridentate, involving the coordination of the isoquinolinic nitrogen (N), the phenolic oxygen (O_phen_), and one oxygen of the carboxylic group (O_carb_).

**Figure 4:**
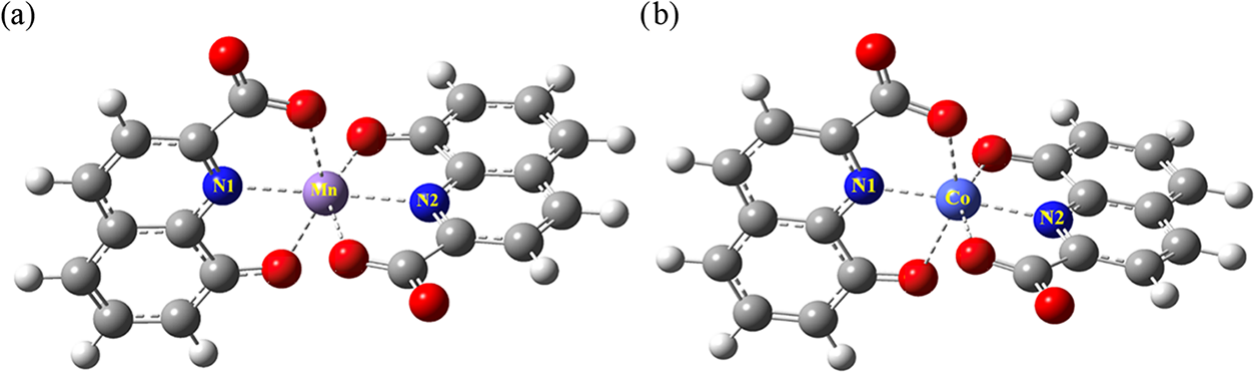
DFT-optimized structure of the (a) MnL_2_^2–^ and (b) CoL_2_^2–^ complexes considering the ligand acting as a tridentate.

These calculations were also performed for the tridentate structure including two further water molecules in the model, to make energetic comparisons with analogous bidentate structures more reliable (the inclusion of two water molecules was necessary in the latter case to guarantee the hexacoordination of the metal center). The resulting tridentate structure (Figure S8a) is more stable (∼32.3 kJ mol^–1^) than the bidentate with 8-HQA coordinating through N and O_phen_, while no optimized structures were obtained considering the coordination through N and O_carb_. Moreover, the most stable MnL_2_^2–^ structure (i.e., the tridentate) shows that the two ligands lie perpendicularly to one another (Figure 4a). The corresponding bond distances from Mn^2+^ are 2.18, 2.21, and 2.30 Å for Mn–N, Mn–O_phen_, and Mn–O_carb_, respectively, and are the same for both coordinated ligands (with differences below 0.01 Å). Similarly, for the CoL_2_^2–^ species, the tridentate structure (with two water molecules considered during calculations, Figure S8b) is more stable than the corresponding bidentate structures by 44.4 and 66.5 kJ mol^–1^ when coordination occurs via O_phen_ or O_carb_, respectively. Even in the case of the most stable tridentate configuration of CoL_2_^2–^ species (Figure 4b), the two ligands are perpendicular to each other, showing the same (always <0.01 Å) bond distances from Co^2+^ of analogous donor atoms: Co–N = 2.01 Å, Co–O_phen_ = 2.14 Å, and Co–O_carb_ = 2.23 Å. Note that a similar orthogonal arrangement of the ligands and similar bond distances between the coordinating atoms and the metal center(s) were also observed by McDonald et al.^33^ in the crystal structures of bis-ligated 8-HQA complexes of Zn^2+^ and Cd^2+^.

In addition to Mn^2+^ and Co^2+^, the structures of the ML_2_^2–^ species of Fe^2+^, Ni^2+^, Cu^2+^, and Zn^2+^ were also optimized, and the main geometrical features are reported in Table S3. The complex configuration obtained is rather similar in all cases under scrutiny, with the two ligands always lying perpendicularly to one another and with the same bond lengths between analogous donor atoms. However, the metal–ligand bond lengths were observed to change across the fourth period, with implications on the complex stability, as discussed in the dedicated section below.

#### 3.5.2 MLH Species

According to previous findings,^14–16,33,48^ when 8-HQA is monoprotonated (i.e., as LH^–^ species), the proton is bound to O_phen_. Quantum mechanical calculations showed that, even when monoprotonated, 8-HQA still acts as tridentate when coordinating Mn^2+^ to form the MLH^+^ species (Figure S9a), with a slightly longer bond distance for Mn–O_phen_ (i.e., 2.47 Å) than Mn–O_carb_ (i.e., 2.09 Å; Mn–N bond is 2.18 Å). This can be expected also considering that the phenolate is protonated and thus uncharged while the carboxylate shares a net negative charge. Interestingly, the MLH^+^ species showed enough room to accommodate either three or four water molecules in the coordination shell (Figure S9a,b), with a preference for the latter (∼16 kJ mol^–1^), although the addition of thermal and entropic effects could likely reduce or revert such a stability difference. Hence, static calculations support the existence of an equilibrium between different solvent configurations in aqueous solution and a relatively fast water exchange between coordinated and bulk water molecules. Note that when the coordination is enhanced by the presence of the fourth water molecule, the bond distances with Mn^2+^ become slightly more elongated (i.e., Mn–N = 2.23 Å, Mn–O_carb_ = 2.17 Å, Mn–O_phen_ = 2.50 Å in the optimized structure with four water molecules–Figure S9b–vs Mn–N = 2.18 Å, Mn–O_carb_ = 2.09 Å, Mn–O_phen_ = 2.47 Å in the optimized structure with three water molecules–Figure S9a). Similar results were observed when considering the optimized structures of the analogue CoLH^+^ species, whose overcoordinated configuration (Figure S10b) is slightly lower in energy (∼3 kJ mol^–1^) than the hexacoordinate (Figure S10a). In the most stable structure in Figure S10b, bond distances are 2.09, 2.51, and 2.04 Å for Co–N, Co–O_phen_, and Co–O_carb_, respectively. Noteworthy, coordination numbers higher than 6 have already been observed for both Mn^2+^ and Co^2+^ complexes (see, e.g., refs 49,50 ).

#### 3.5.3 ML Species

Finally, for the ML species, quantum mechanical calculations showed that the tridentate configuration is the most stable for both Mn^2+^ and Co^2+^. The optimized structures are shown in Figure S11. In terms of the bond distances with the metal centers, we noted that 8-HQA in the monoligated species (i.e., ML) is significantly closer than in the bis-ligated (i.e., ML_2_^2–^) species discussed above (for ML, it is Mn–N = 2.15 Å, Mn–O _carb_ = 2.18 Å, and Mn–O_phen_ = 2.24 Å; Co–N = 1.98 Å, Co–O_carb_ = 2.08 Å, and Co–O_phen_ = 2.18 Å). However, in this case, the M–O_phen_ distance appeared noticeably shorter (Δ ∼ 0.3 Å) than in the corresponding monocoordinated MLH^+^ species displaying a protonated ligand, as expected due to the enhanced interaction between the metal ion and the phenolate oxygen (i.e., Mn–O_phen_ = 2.24 Å in ML and 2.50 Å in MLH^+^). In other words, when the phenolate is deprotonated (as in ML species), the electrostatic contribution of the net negative charge of O_phen_ allows the M–O_phen_ bond distances to be significantly reduced in both Mn^2+^ and Co^2+^ complexes compared to that of the analogue MLH^+^ with protonated O_phen_. In analogy with the MLH^+^ species, the ML species showed the possibility of both hexa- and heptacoordination of the central metal with three or four water molecules, respectively, in addition to the ligand. The octahedral complex configuration appeared more stable by ∼5 kJ mol^–1^ than the 7-fold configuration, in contrast to the previous case (i.e., MLH^+^), due to the stronger metal–ligand interaction in ML, thus suggesting again the formation of different structural arrangements in solution.

### 3.6 Stability Constants vs M–N Bond Length

The stability constants of the metal complexes formed between 8-HQA and the investigated divalent cations, in particular ML_2_^2–^ species, increase throughout the series from Mn^2+^ to Cu^2+^ and then slightly decrease for Zn^2+^, following the general trend Mn^2+^ < Fe^2+^ < Co^2+^ < Ni^2+^ < Cu^2+^ > Zn^2+^, which is in agreement with the Irving–Williams series. The stability of the formed complexes strongly depends on the metal cation and can be explained by its chemical properties, such as ionic radius and charge density. Based on the structure of 8-HQA, it is clear that the quinolinic nitrogen plays a crucial role in coordination during metal complex formation. As such, the stability constants of the formed metal complexes are likely closely related to the M–N bond length.^51^ Generally, as this length shortens, the metal–ligand interaction strengthens due to better orbital overlapping and enhanced electrostatic attraction, resulting in higher stability constants according to the Irving–Williams series. To prove this concept even for 8-HQA, the structures of the ML_2_^2–^ species for all divalent cations of the series were optimized by quantum mechanical calculations. The corresponding average M–N bond lengths (Table S3) are then plotted in Figure 5 for each cation, along with the stability constants of the corresponding species (reversed axis), showing that both follow the same trend (i.e., shorter length ≡ higher stability constant) postulated by the Irving–Williams series.

**Figure 5:**
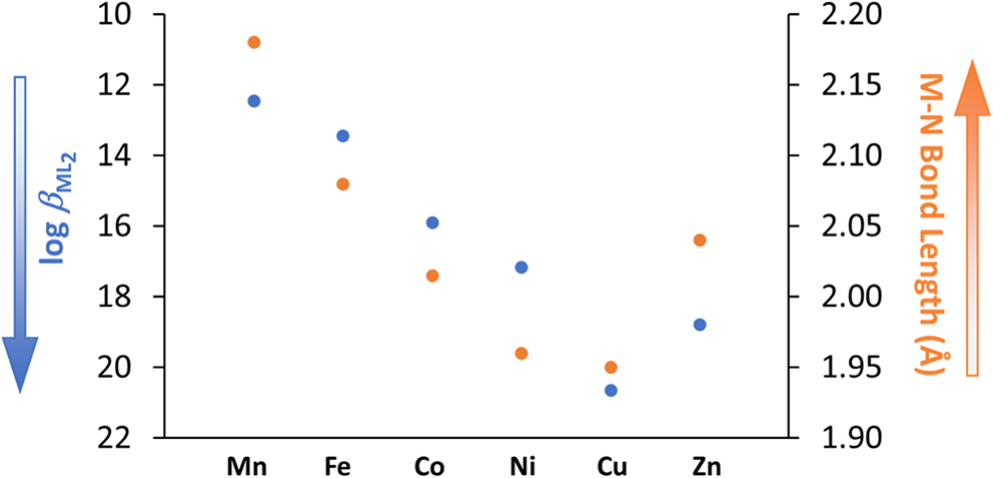
Stability constants and average M–N bond lengths (Å) of the optimized structures of ML_2_^2–^ species of divalent cations of the Irving–Williams series.

### 3.7 Chemical Speciation and Sequestering Ability

The different stability of various M^2+^/8-HQA complexes (Table 1) obviously affects the chemical speciation of the investigated cations in aqueous solution in the presence of 8-HQA. The speciation plots, drawn through PyES software,^29^ are depicted in Figures 6 (and S12 to S16). It is worth mentioning that all speciation diagrams were drawn for the same conditions (*c*_L_ = 10^–4^ mol dm^–3^), an intermediate order of magnitude between total concentrations used during potentiometric (*c*_L_ ∼ 10^–3^ mol dm^–3^) and UV–vis spectrophotometric (*c*_L_ ∼ 10^–5^ mol dm^–3^) measurements, for a better comparison between various systems.

**Figure 6:**
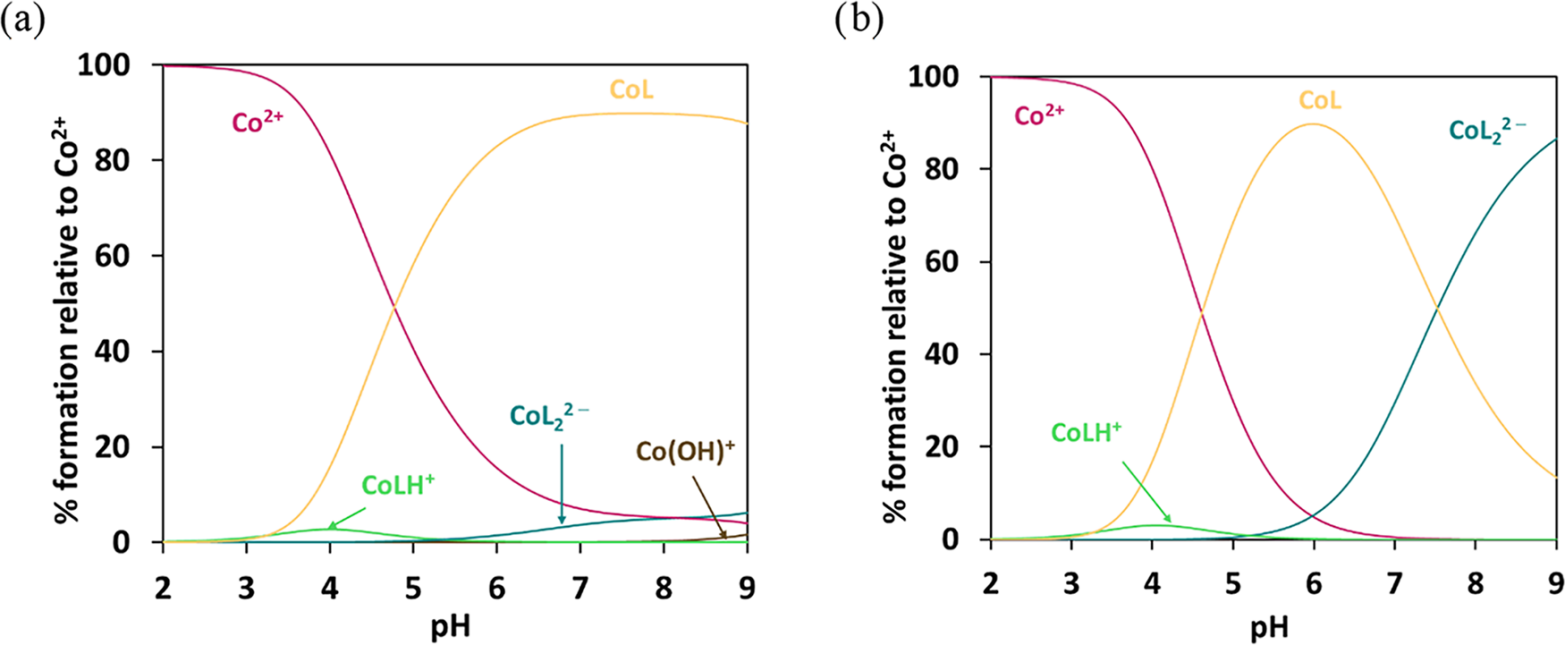
Distribution diagrams of Co*_p_* L*_q_* H*_r_* species as a function of pH in the Co^2+^/8-HQA system in KCl_(aq)_ at *I* = 0.2 mol dm^–3^ and *T* = 298.2 K. (a) *c*_L_ = *c*_Co_ = 10^–4^ mol dm^–3^ and (b) *c*_L_ = 2 × *c*_Co_ = 10^–4^ mol dm^–3^.

The first observation that can be made concerns the formation of the ML_2_^2–^ species that is, as expected, strongly influenced by the *c*_L_/*c*_M_ ratios. In particular, by the comparison of Figure 6a,6b, when *c*_L_/*c*_M_ = 1:1, the speciation of Co^2+^ shows the formation of CoL as the main complex above pH ∼ 4, reaching a maximum of ∼90% at pH ∼ 6.5. However, speciation changes significantly when *c*_L_/*c*_M_ = 2:1, in which the CoL species decreases from pH ∼ 6 in favor of the ML_2_^2–^ complex, CoL_2_^2–^, present at ∼85% at pH ∼ 9. Different behavior is observed for the Fe^2+^/8-HQA system, where even at *c*_L_/*c*_M_ = 2:1, the FeL_2_^2–^ species remains below 10% in the conditions of Figure S13b.

Since the formation of ML_2_^2–^ species generally becomes significant at pH > ∼5, the chemical speciation of all of the investigated systems below this pH value remains almost independent of the *c*_L_/*c*_M_ ratio, as only the mononuclear ML and MLH^+^ species form in percentages slightly affected by that ratio. For example, in the case of Cu^2+^/8-HQA (Figure S14), the formation percentage of CuL species only varies by ∼10% shifting from *c*_L_/*c*_M_ = 1:1 (∼70%) to *c*_L_/*c*_M_ = 2:1 (∼80%). For Ni^2+^ (Figure S14) and Zn^2+^ (Figure S16), these differences are even smaller.

Another interesting aspect emerging from the analysis of the speciation diagrams reported in Figures is that 8-HQA strongly binds all the investigated cations over a wide pH range through the formation of the ML, MLH^+^, and ML_2_^2–^ species. As a result, the formation of hydrolytic species is negligible for all divalent cations except in the case of Zn^2+^, where Zn­(OH)_2_ reaches a formation of ∼40% at pH ∼ 9 (Figure S16). Nevertheless, it is important to point out that considering such species in the speciation model during data analysis and calculations through an accurate set of hydrolysis constants is still fundamental. A detailed discussion on this topic lies outside the scope of this article, but some comments are given as Supporting Information.

The above-mentioned significant binding ability of 8-HQA makes this ligand a promising sequestering agent toward the investigated cations. However, it has been frequently pointed out that the assessment of the sequestering ability of a ligand toward various metal ions is not easily accessible through the simple comparison of the stability constants of metal/ligand complexes, especially if the chemical speciation of systems to compare is different (i.e., different species formed, different acid–base properties of both cations and ligands).^52–57^ An efficient method for this purpose is the use of *ad-hoc* defined parameters, like the pL_0.5_, which represents the total ligand concentration (as −log *c*_L_) needed to complex half of the metal cation of interest (present at the lowest possible concentration allowed by software; in our case: *c*_M_ = 10^–23^ mol dm^–3^) under given conditions of the investigated system.^55,57^ Accordingly, the higher is the pL_0.5_, the higher is the sequestering ability. Analyzing the calculated values of pL_0.5_, graphically represented in Figure 7 for a more visual evaluation, we can say that 8-HQA has a great preference for Cu^2+^ in comparison with the other metal cations of the Irving–Williams series (see Table S4 for further details). Furthermore, differently from highly hydrolyzable cations like Fe^3+^ and Ga^3+^ (in which hydrolysis competes with ligand sequestration when increasing pH), for all the divalent cations of this study, we observe that the sequestering ability of 8-HQA almost linearly increases with pH (Figure S17) up to pH ∼ 8, when hydrolytic species compete more significantly with the ligand complexation. This can be easily justified by the formation of the ML_2_^2–^ species, whose contribution is more relevant at higher pH values, which strongly inhibits cation hydrolysis.

**Figure 7:**
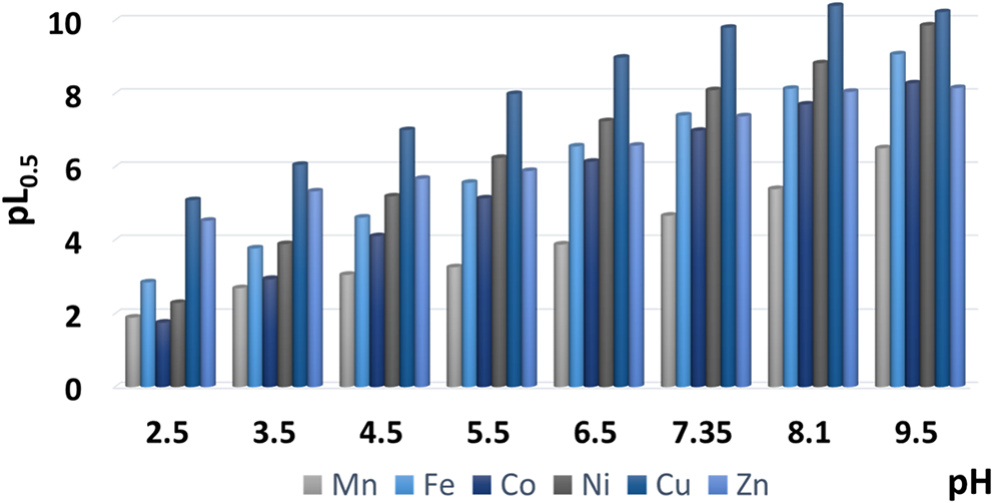
Sequestering ability (pL_0.5_) of 8-HQA toward M^2+^ (M = Mn, Fe, Co, Ni, Cu, Zn), as a function of pH, in KCl_(aq)_ at *I* = 0.2 mol dm^–3^ and *T* = 298.2 K.

## 4 Conclusions

In the framework of a series of studies dedicated to the evaluation of the chelating ability of the tryptophan metabolite, 8-hydroxyquinoline-2-carboxylic acid (8-HQA, LH_2_), toward biological relevant metal cations, in this article, we investigated the coordination mode and the chemical speciation in aqueous solution of M^2+^/8-HQA systems, being M^2+^ the metal cations of the Irving–Williams series, i.e., Mn^2+^, Fe^2+^, Co^2+^, Ni^2+^, Cu^2+^, and Zn^2+^. We have found that 1:1 (MLH^+^, ML) and 2:1 (ML_2_^2–^) species are formed in all systems over a wide pH range. At pH relevant for many natural waters and biological fluids, the main species is the ML_2_^2–^, whose formation is highly dependent on the *c*_L_/*c*_M_ ratio. Metal complexation by 8-HQA is strong enough to significantly affect metal ion speciation, especially in relation to the suppression of cations’ hydrolysis (and precipitation). Together with the determination of the stability constants of the formed metal complexes, differential pulse anodic stripping voltammetry (DP-ASV) and cyclic voltammetry (CV), EPR (electron paramagnetic resonance spectroscopy), and quantum mechanical calculations have also been used to derive information about the coordination modes, structure, and nature of formed species. DP-ASV and CV experiments supported the proposed chemical speciation model, in particular, in relation to the formation of the bis-ligated ML_2_^2–^ species over the possible ML­(OH)^−^. This is also in line with CW-EPR results, which further showed that the investigated metal cations occur in their high-spin state in the formed complexes. Quantum mechanical calculations proved the involvement of all three binding sites of 8-HQA in metal coordination; i.e., the ligand behaves as a tridentate chelator through the isoquinolinic nitrogen (N), the phenolic oxygen (O_phen_), and one oxygen of the carboxylic group (O_carb_). This is likely to happen even in the monoprotonated MLH^+^ species, where the protonated O_phen_ is still involved in the coordination. These studies further provided, for MLH^+^ and ML species, the possible coexistence of distinct solvent configurations in aqueous solution, with three or four water molecules in the first coordination sphere of the metal center. Furthermore, these calculations proved the correlation between the stability of metal complexes (in particular for ML_2_^2–^) and the M–N bond length, which obviously decreases with the increase of the stability constants of the metal complexes. Overall, results show that 8-HQA complexation toward the divalent metal ions in the study follows the known Irving–Williams series trend. In particular, log β_120_ for ML_2_^2–^ species increases from Mn^2+^ to Cu^2+^ and then slightly decreases for Zn^2+^. Finally, the sequestering ability of 8-HQA toward the studied metal cations was quantified by the calculation of several pL_0.5_ at various pH, demonstrating that this ligand is a good chelator in various pH conditions. All the results obtained suggest a possible role of 8-HQA in microbial metal scavenging and microbiota regulation via the so-called nutritional immunity strategy, since it forms stable metal complexes with metals that are likely to be present in the environment where 8-HQA, tryptophan, and its metabolites act, in particular in the gut. With detailed knowledge of the chemical speciation of 8-HQA in the presence of distinct cations, further biological studies could be proposed to better understand and confirm the biological role of such an interesting ligand.

## Supplementary Material



## Notes

Tables with further data (other thermodynamic data, bond lengths, and pL_0.5_), additional experimental details, and supplementary figures for more comprehensive knowledge of the different systems (PDF)
