# Supplementary material for: Chemical Speciation and Coordination Behavior of 8‑Hydroxyquinoline-2-carboxylic Acid with Divalent Cations in Aqueous Solution: An Irving–Williams Series Study
Source: ACS Omega. 2025 Nov 26;10(48):58588–99. doi: 10.1021/acsomega.5c06622 (PMC12771187; doi:10.1021/acsomega.5c06622)
Supplement: Supplementary file 1 [file ao5c06622_si_001.pdf]

SUPPORTING INFORMATION

Chemical Speciation and Coordination Behavior of 8-Hydroxyquinoline-2-Carboxylic Acid with Divalent Cations in Aqueous Solution: An Irving–Williams Series Study

Anna Baryłka,[a] Rafał Bukrym,[b] Izabela Ryza,[a] Clemente Bretti,[c] Sourab Sinha,[d] Rosita Cappai,[e] Gabriele Lando,[c] Oluseun Akintola,[f] Winfried Plass,[f] Beata Godlewska-Żyłkiewicz,[b] Giuseppe Brancato,[d,g] Demetrio Milea,[c,*] Sofia Gama[h,*]

[a] Doctoral School, University of Bialystok, K. Ciolkowskiego 1K, 15-245 Bialystok, Poland.

[b] Department of Analytical and Inorganic Chemistry, Faculty of Chemistry, University of Bialystok, K. Ciolkowskiego 1K, 15-245 Bialystok, Poland.

[c] Dipartimento di Scienze Chimiche, Biologiche, Farmaceutiche ed Ambientali, CHIBIOFARAM, Università degli Studi di Messina, Viale F. Stagno d'Alcontres 31, 98166 Messina, Italy.

[d] Scuola Normale Superiore e CSGI, Piazza dei Cavalieri, 7, 56126, Pisa, Italy.

[e] Dipartimento di Scienze Chimiche, Fisiche, Matematiche e Naturali, Università di Sassari, via Vienna 2, Sassari, 07100, Italy.

[f] Institut für Anorganische und Analytische Chemie Friedrich-Schiller-Universität Jena, Humboldtstr. 8, 07743 Jena, Germany.

[g] Istituto Nazionale di Fisica Nucleare (INFN), Largo Pontecorvo, 3, 56127 Pisa, Italy.

[h] Centro de Ciências e Tecnologias Nucleares, Instituto Superior Técnico, Universidade de Lisboa, Estrada Nacional 10 (km 139.7), 2695-066, Bobadela LRS, Portugal.

* Corresponding and co-last authors: dmilea@unime.it (Demetrio Milea); sofia.gama@ctn.tecnico.ulisboa.pt (Sofia Gama).

Stability constants and speciation model

Table S1. Protonation constants of 8-HQA (LH_2) determined by ISE-H^+ potentiometric measurements at $T = 298.2 \pm 0.1$ K and $I = 0.2$ mol dm^{-3} in $KCl_{(aq)}$.

**Equilibrium**	**log K_i[a]**
$H^+ + L^{2-} \rightleftarrows LH^-$	9.56
$H^+ + LH^- \rightleftarrows LH_2$	3.96

[a] log K_i refer to equilibrium: $H + LH_{i-1} = LH_i$; from ref.[1]

Table S2. Hydrolysis constants of studied metal cations calculated at $T = 298.2$ K and $I = 0.2$ mol dm^{-3} in $KCl_{(aq)}$.

**Equilibrium**	**log β[a]**					
	**Mn^{2+}**	**Fe^{2+}**	**Co^{2+}**	**Ni^{2+}**	**Cu^{2+}**	**Zn^{2+}**
$M^{2+} + H_2O \rightleftarrows MOH^+ + H^+$	-10.63	-9.71	-9.39	-10.02	-8.42	-9.2
$M^{2+} + 2\ H_2O \rightleftarrows M(OH)_2 + 2\ H^+$	-22.45	-20.82	-18.78	-21.54	-17.73	-17.16
$M^{2+} + 3\ H_2O \rightleftarrows M(OH)_3^- + 3\ H^+$	-35.0	-31.01	-31.51	--	-27.81	-28.41
$M^{2+} + 4\ H_2O \rightleftarrows M(OH)_4^{2-} + 4\ H^+$	-47.79	-45.54	-45.78	--	-38.67	-40.66
$2\ M^{2+} + H_2O \rightleftarrows M_2(OH)^{3+} + H^+$	-10.44	--	--	-10.36	--	-8.77
$2\ M^{2+} + 2\ H_2O \rightleftarrows M_2(OH)_2^{2+} + 2\ H^+$	--	--	--	--	-10.82	--
$2\ M^{2+} + 3\ H_2O \rightleftarrows M_2(OH)_3^+ + 3\ H^+$	-23.9	--	--	--	--	--
$2\ M^{2+} + 6\ H_2O \rightleftarrows M_2(OH)_6^{2-} + 6\ H^+$	--	--	--	--	--	-57.52
$4\ M^{2+} + 4\ H_2O \rightleftarrows M_4(OH)_4^{4+} + 4\ H^+$	--	--	--	-27.25	--	--

[a] from ref. [2-3]

Comments on the inclusion or not of hydrolysis constants in calculations

As a premise, this discussion is simplified to the outmost, and it is done just to clarify the process behind data analysis for the determination of the stability constants to non-experts in the field, and to stress the importance of the inclusion of accurate hydrolysis (and other) constants in input files during software elaborations.

The determination of the stability constants of metal complexes is nowadays mainly performed by nonlinear least square minimization software able to fit experimental data (*e.g.*, potentiometric, spectrophotometric or NMR titrations) according to a chemical speciation model, proposed by the investigator. Strategies adopted by most common software usually (but not exclusively) imply the solution of mass balance equations (MBE), in which the analytical concentrations of the components of the chemical system in study are generally given as input, and the free concentrations of various species in MBE are expressed as a function of their stability constants. These constants may be known, or unknown for some species. Noteworthy, one (or more) quantities in MBE may be experimentally measured (*e.g.*, the free proton concentration during acid-base titrations), reducing the unknowns, but this may not necessarily be the case (*e.g.*, some UV/Vis spectra). Finally, once all available data (*e.g.*, stoichiometry and number of species included in the model through their stability constants; analytical concentrations, etc.) are given as input, software uses them to reproduce the experimental data (*e.g.*, acid-base titration curves) and, through iterative methods, refine all the adjustable parameters (like the unknown stability constants to determine) until the difference between calculated and experimental data is minimized.

As such, the inclusion or not of a species in the chemical speciation model unavoidably affects the formation of other species and, most importantly, influences the numerical value of their refined stability constants. To be clearer, the optimized value of a given stability constant is dependent on the values of other stability constants included in the model independently of the fact that a given species is effectively formed or not. Without entering into equilibrium analysis discussions and calculations (that can be also found in basic analytical chemistry textbooks), this can be easily verified directly during experimental data analysis, simply running software modifying the chemical speciation model used as input, either changing the values of the stability constants or neglecting some species.

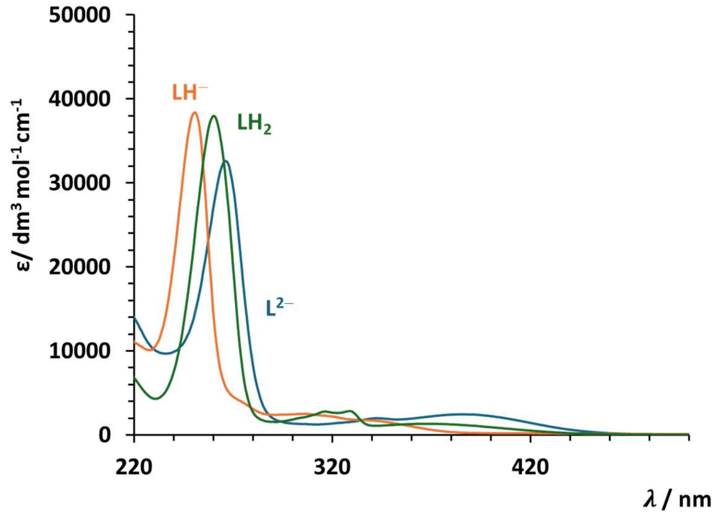

Figure S1. Molar absorptivity spectra of LH_2, LH^- and L^{2-} species. Ref.[4]

a) b)

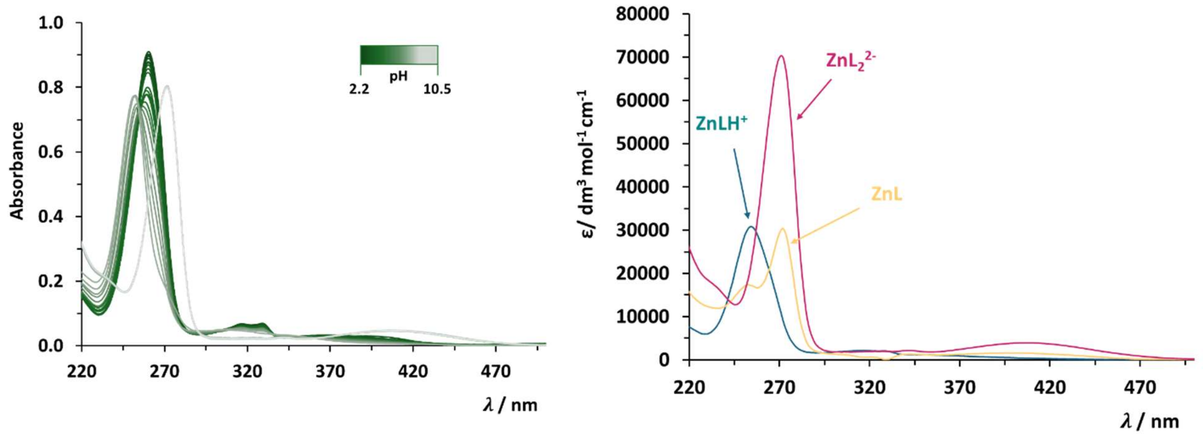

Figure S2. (a) Experimental UV-Vis spectra of Zn^{2+}/8-HQA system at 1:2 ratio, measured at different pH values, $c_L = 2.62 \times 10^{-5}$ mol dm^{-3}, in $I = 0.2$ mol dm^{-3} $KCl_{(aq)}$ and $T = 298.2$ K, (b) calculated molar absorptivity spectra of $ZnLH^+$, ZnL_2^{2-} and ZnL species.

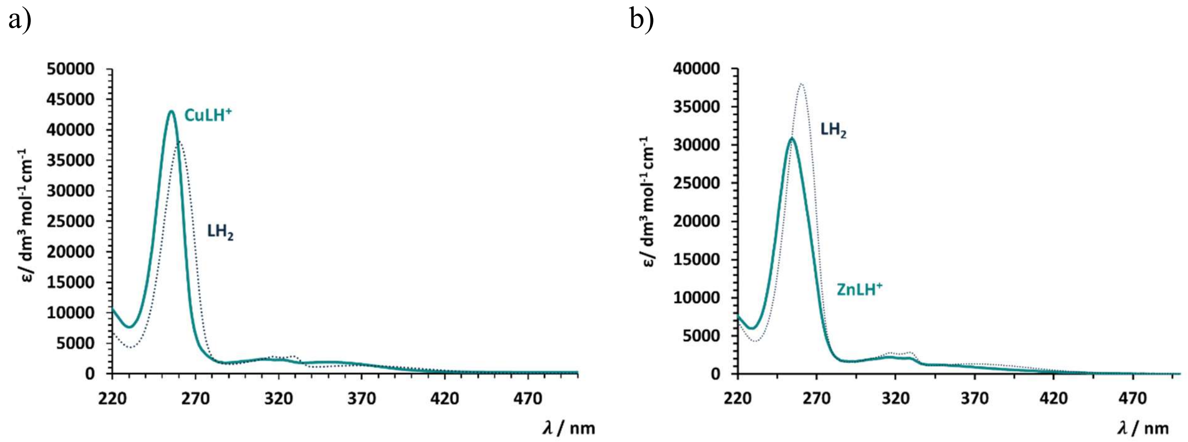

Figure S3. Calculated molar absorptivity spectra of a) $CuLH^+$ and b) $ZnLH^+$, overlapped with the molar absorptivity spectra of LH_2.

Further details on voltammetric experiments

The linearity of the response both in the DP-ASV and CV was tested in the range 1×10^{-6} to 1×10^{-4} mol dm^{-3} of total manganese concentration ($R^2 = 0.991$). A single peak located at –1.5 V is obtained, which linearly increases with concentration, but it is featured by a quite uncommon width at half height of ~ 100 mV, as well as a significant asymmetry. The CV recorded on a manganese solution (Figure S4) showed that the cathodic peak is far lower than the anodic. This applies both at neutral and alkaline pH, indicating a quasi-reversible / irreversible nature of the electrochemical process and also explaining the width at half height value (~ 85 − 110 mV) of the peaks in DP-ASV.

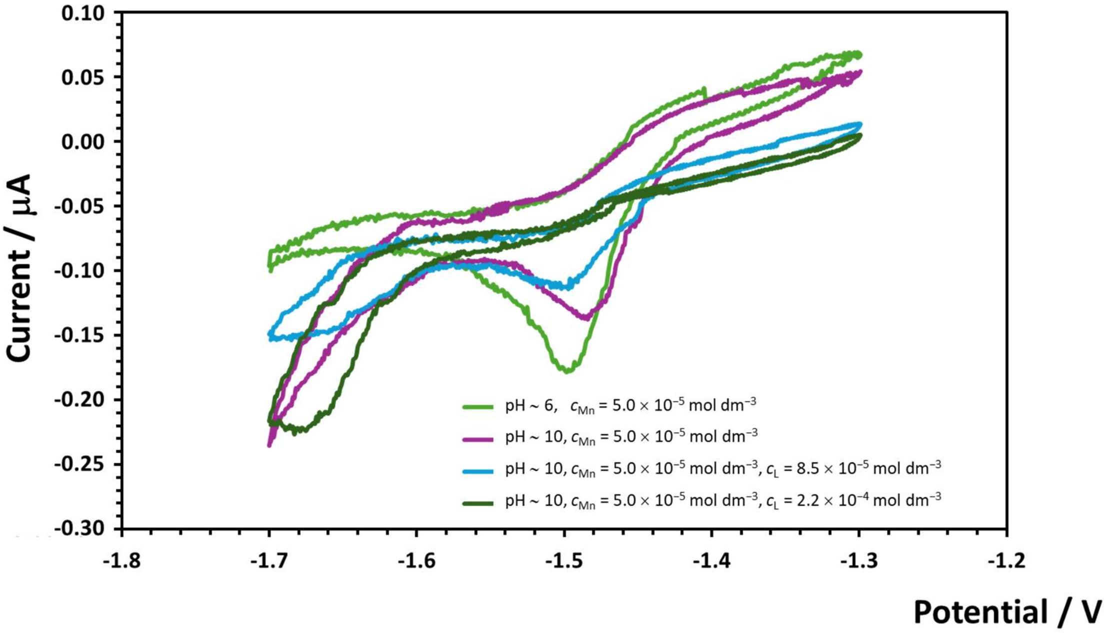

Figure S4. Examples of cyclic voltammograms of Mn^{2+} solutions ($c_{Mn} = 5 \times 10^{-5}$ mol dm^{-3}) at pH ~ 6 (━) and pH ~ 10 (━), and at pH ~ 10 in the presence of different concentrations of 8-HQA: $c_L = 8.5 \times 10^{-5}$ mol dm^{-3} (━) and $c_L = 2.2 \times 10^{-4}$ mol dm^{-3} (━).

In a typical titration performed in an acid-base feature, the first point was recorded with only metal cation and the corresponding counter-ions, to obtain the peak height and position of the parent peak, in a condition where the metal cation was free from any interaction with the ligand and hydroxide ion, as the ligand was not present and the pH was too low to consider the possible formation of Mn^{2+}/OH^- species. Successively, the ligand was added to reach the desired pH (generally ~ 4) and $c_L:c_M$ ratio. Then, a standard solution of strong base ($KOH_{(aq)}$) was added to perform a full acid-base titration. An example of such titration, relative to that discussed in the main text, is given: the first point of the titration was recorded at pH = 6.25 with only Mn^{2+} (c_{Mn} = 4×10^{-5} mol dm^{-3}), K^+ and Cl^- in solution. In those conditions, the peak height and position were 0.26 μA and −1.495 V, respectively. Successively, a total of six additions of the 8-HQA solution were done before reaching the desired pH (3.69) and $c_L:c_M$ ratio (14.2), recording a voltammogram after each addition. Then, aliquots of standardized $KOH_{(aq)}$ were added to the solution collecting 25 further voltammograms, for a total of 32 points per titration, in the pH range comprised between 3.69 and 10.92.

A decrease in the peak height and a small shift to more negative values in the peak position is observed after the first addition of 8-HQA. At pH > 3.85 a shoulder appears in the left part of the peak and the diffusion current further decreases, as an indication of the coexistence of labile species and non-labile species. Upon increasing pH, the shoulder gradually develops into a second peak, which eventually become fully separated at pH ~ 9. Such a behavior is typical of the coexistence of both labile and non-labile species.[5] Three full voltametric titrations have been performed at fixed $c_L:c_M$ ratios, ranging between 5 and 15, as a function of pH.

Ligand titrations started exactly in the same way as the acid-base titrations, namely with only Mn^{2+}, K^+ and Cl^- in solution. In this case, the standard base was added first to reach an alkaline

pH value (> 9.5) and several additions of ligand, up to $c_L = 2 \times 10^{-4}$ mol dm^{-3}, were provided. From an electrochemical point of view, the behavior of the metal peak is very similar (*i.e.*, shoulder formation, peak height decreasing) to that discussed for acid-base titrations, even though the shifts observed in the peak position were larger.

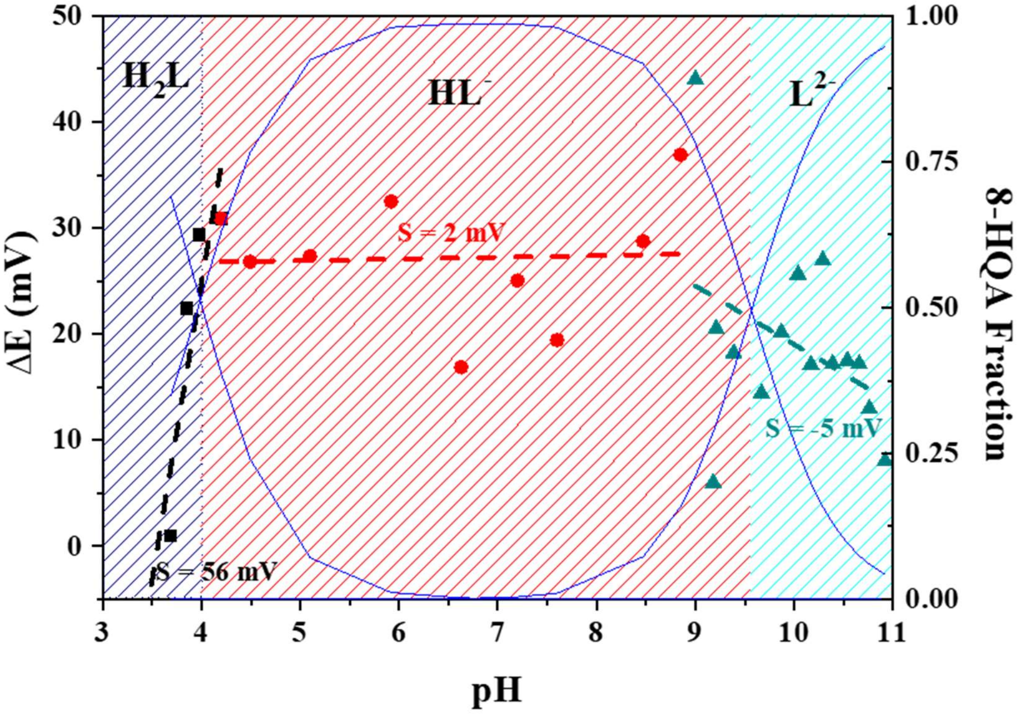

Figure S5. Superimposition of the ligand distribution diagram (lines and regions evidenced) for Mn^{2+} and the plot of metal reduction peak shift (ΔE, mV) *vs*. pH in an acid-base titration at a fixed ratio of c_L:c_M = 14.2.

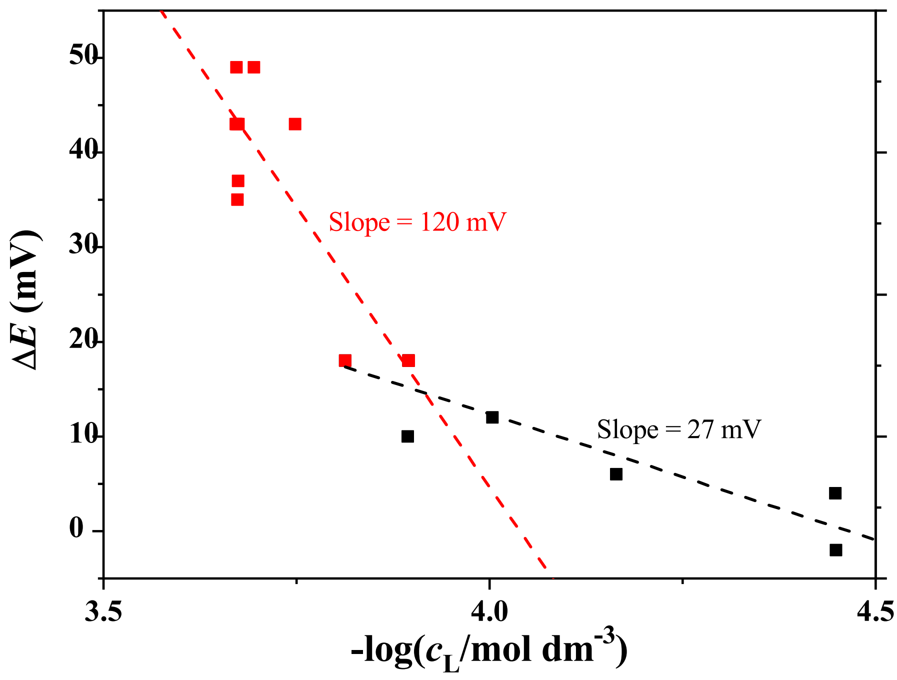

Figure S6. ΔE (ΔE = E_{parent} – E_{labile}) *vs*. -log (c_L / mol dm^{-3}) for Mn^{2+} measured in alkaline conditions (pH ~ 10) upon successive ligand additions at c_M = 1 μmol dm^{-3}, T = 298.2 K and I = 0.2 mol dm^{-3} in $KCl_{(aq)}$.

Electron Paramagnetic Resonance Spectroscopy

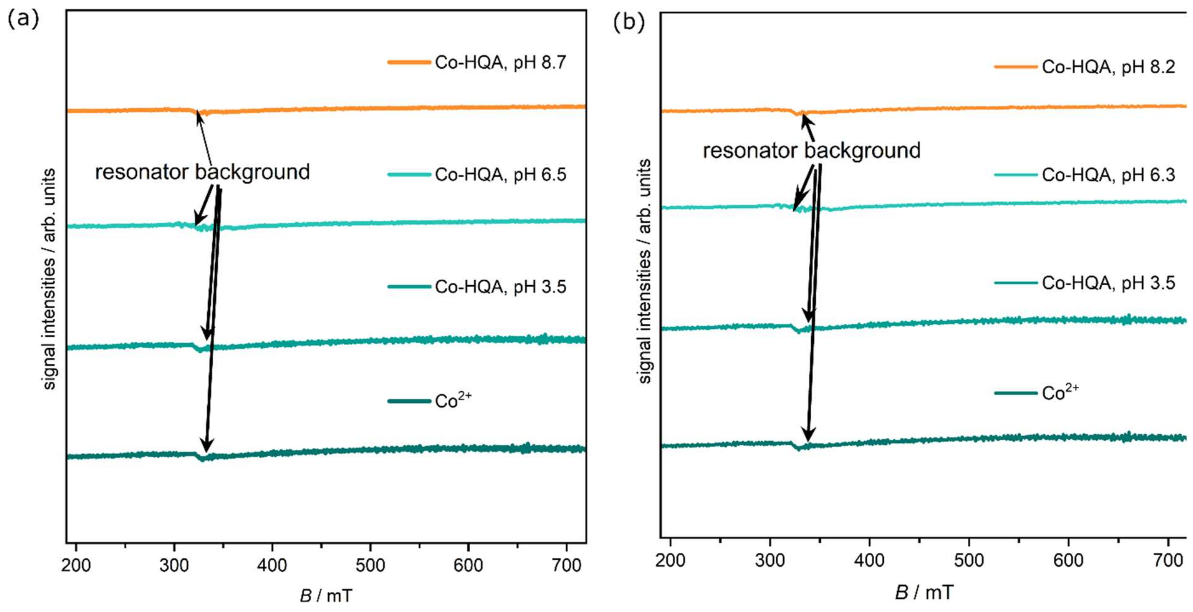

Figure S7. a) EPR spectra of Co^{2+}/8-HQA system $T \sim 90$ K. a) c_M:c_L = 1:1 ($c_M = c_L = 1.2$ mmol dm^{-3}), pH = 3.6, 6.3, and 8.7; (b) c_M:c_L = 1:2 ($c_M = 0.6$ mmol dm^{-3}, $c_L = 1.2$ mmol dm^{-3}), pH = 3.6, 6.3, and 8.2; both include the EPR spectra of Co^{2+} aquo complex (without 8-HQA) at pH ~ 2.

Quantum mechanical calculations

a) b)

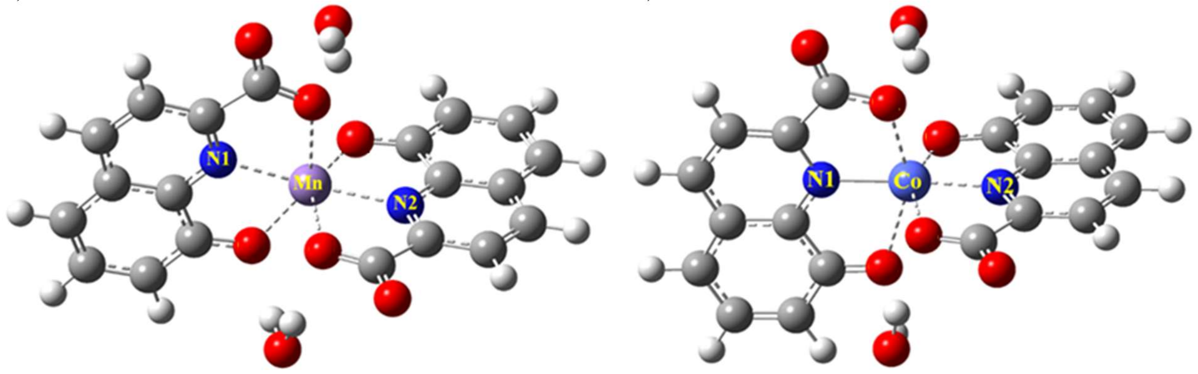

Figure S8. DFT optimized structure of the a) MnL_2^{2-} and b) CoL_2^{2-} complexes considering the ligand acting as a tridentate and including two water molecules in calculations.

a) b)

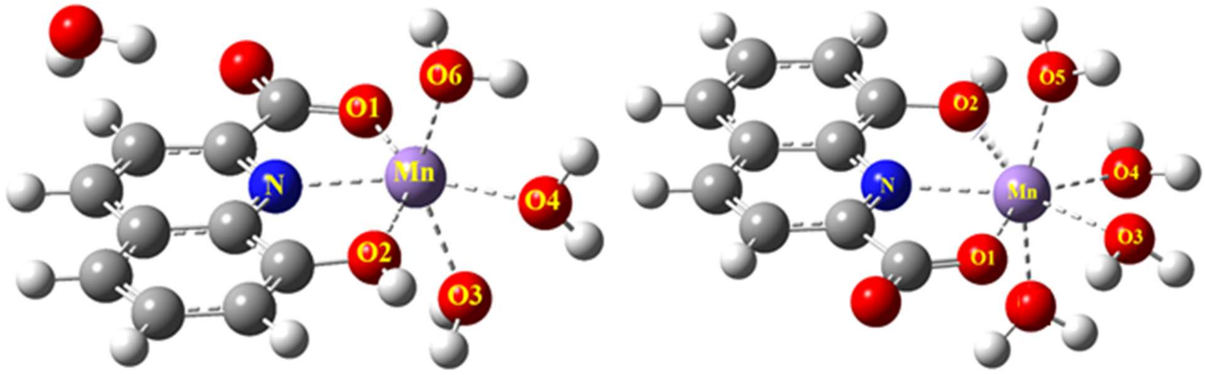

Figure S9. DFT optimized structures of the $MnLH^+$ complex considering the ligand acting as a tridentate chelating agent. a) Three or b) four coordinated water molecules were considered to complete the coordination shell.

a) b)

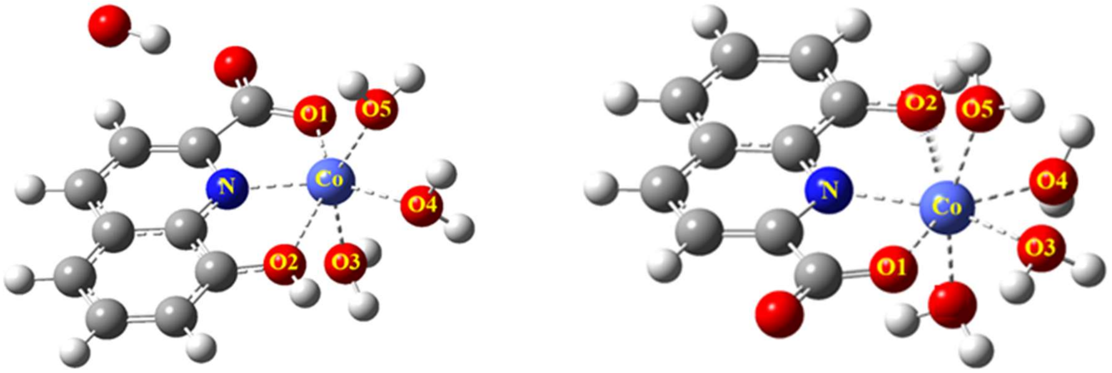

Figure S10. DFT optimized structures of the $CoLH^{+}$ complex considering the ligand acting as a tridentate chelating agent. a) Three or b) four coordinated water molecules were considered to complete the coordination shell.

a) b)

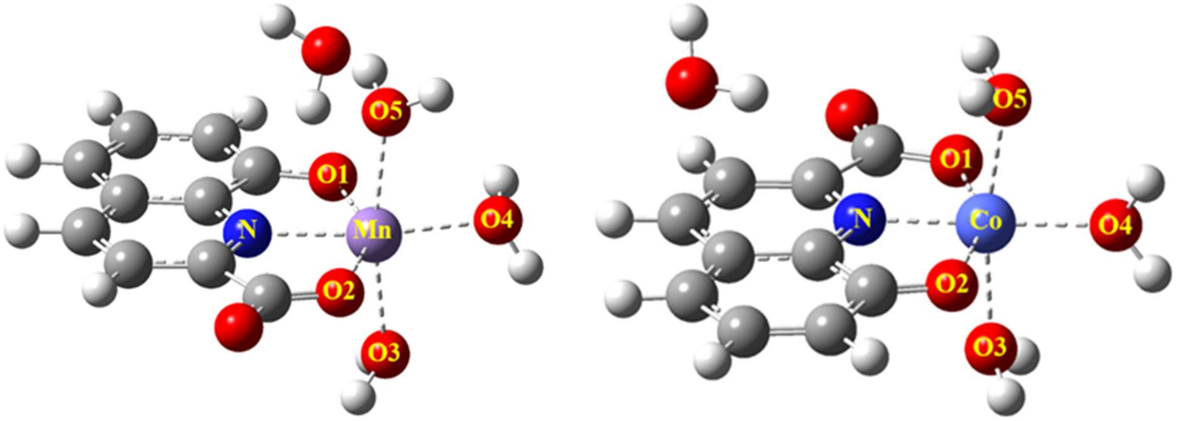

Figure S11. DFT optimized structures of the a) MnL and b) CoL complexes considering the ligand acting as a tridentate chelating agent. In both cases, three water molecules were considered to complete the coordination shell, however also four water molecules could be accommodated in the first coordination shell as seen for the MLH^{+} species (data not shown).

Stability constants vs M–N bond length

Table S3. Stability constants and bond lengths (Å) of the optimized structures of ML_2^{2-} species of the divalent cations of the Irving-Williams series.

**Complexes**	**log β_{120}**	**Bond Length (Å)**		
		M–O_{phen}	M–O_{carb}	M–N
**MnL_2^{2-}**	12.45	2.21	2.30	2.18
**FeL_2^{2-}**	13.45	2.14	2.27	2.08
**CoL_2^{2-}**	15.90	2.14	2.23	2.01
**NiL_2^{2-}**	17.17	2.11	2.32	1.96
**CuL_2^{2-}**	20.64	2.21	2.28	1.95
**ZnL_2^{2-}**	18.78	2.16	2.32	2.04

Chemical speciation and sequestering ability

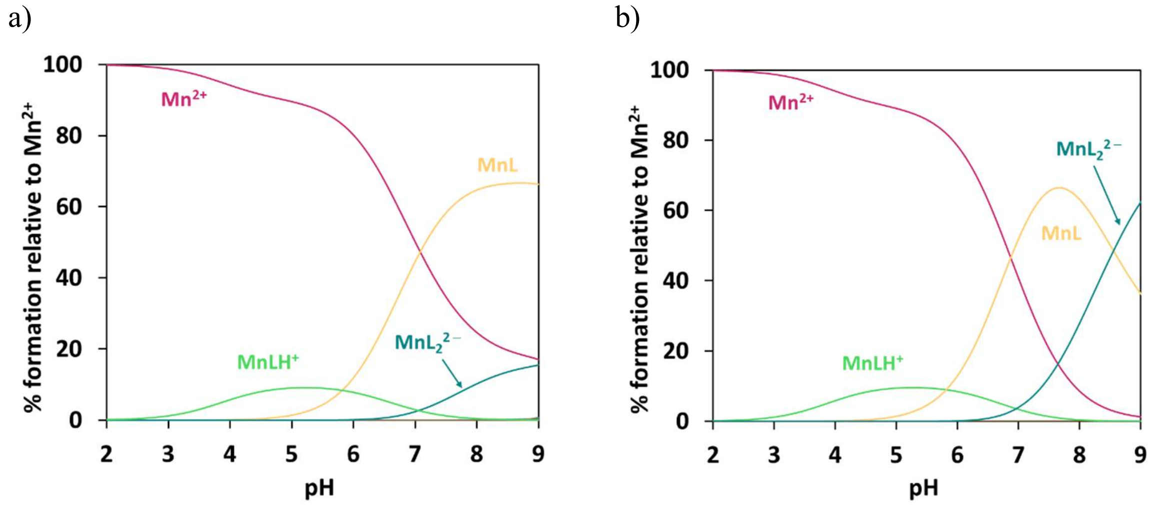

Figure S12. Distribution diagrams of $Mn_pL_qH_r$ species as a function of pH in the Mn^{2+}/8-HQA system in $KCl_{(aq)}$ at $I = 0.2$ mol dm^{-3} and $T = 298.2$ K. a) $c_L = c_{Mn} = 10^{-4}$ mol dm^{-3} and b) $c_L = 2 \times c_{Mn} = 10^{-4}$ mol dm^{-3}.

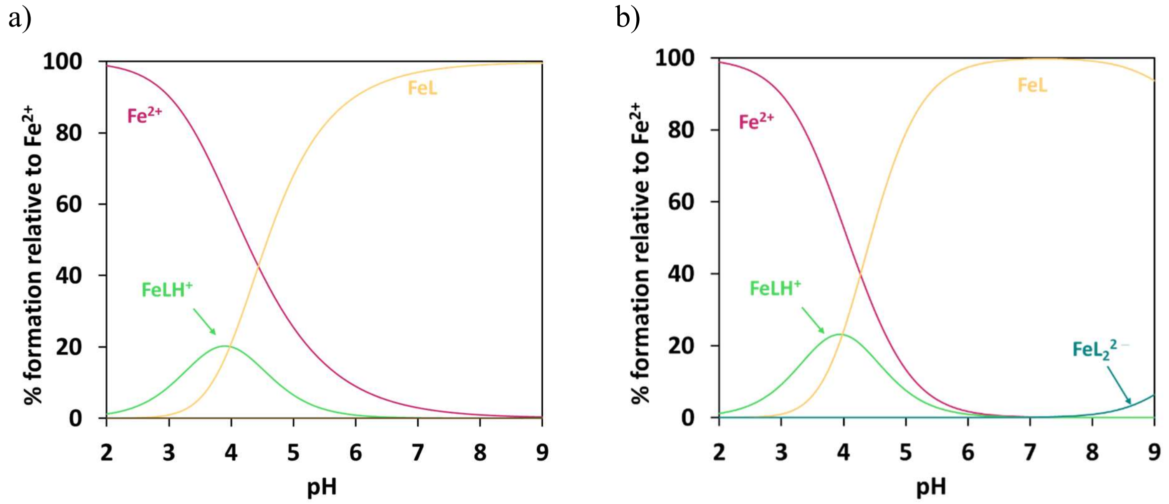

Figure S13. Distribution diagrams of $Fe_pL_qH_r$ species as a function of pH in the Fe^{2+}/8-HQA system in $KCl_{(aq)}$ at $I = 0.2$ mol dm^{-3} and $T = 298.2$ K. a) $c_L = c_{Fe} = 10^{-4}$ mol dm^{-3} and b) $c_L = 2 \times c_{Fe} = 10^{-4}$ mol dm^{-3}.

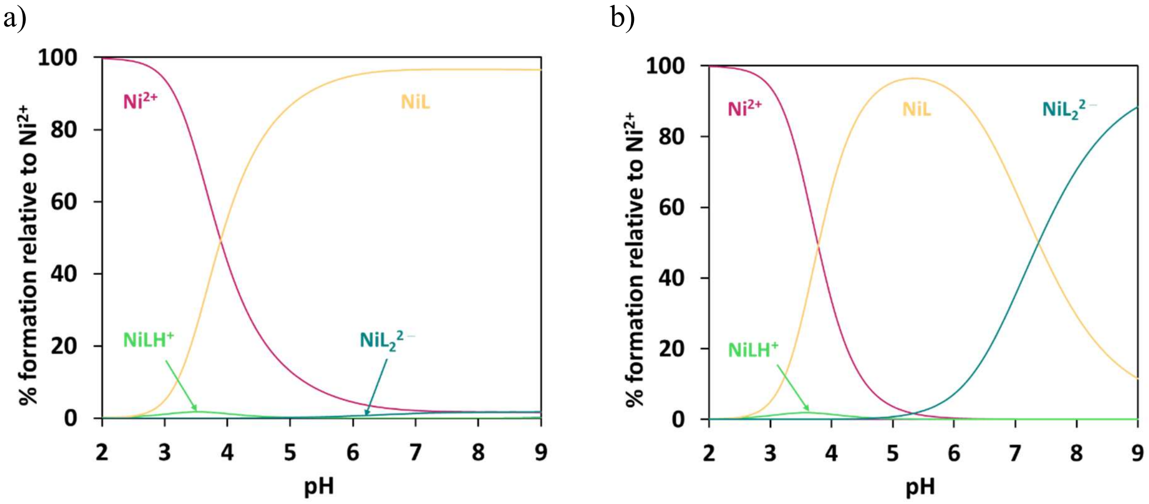

Figure S14. Distribution diagrams of $Ni_pL_qH_r$ species as a function of pH in the Ni^{2+}/8-HQA system in $KCl_{(aq)}$ at $I = 0.2$ mol dm^{-3} and $T = 298.2$ K. a) $c_L = c_{Ni} = 10^{-4}$ mol dm^{-3} and b) $c_L = 2 \times c_{Ni} = 10^{-4}$ mol dm^{-3}.

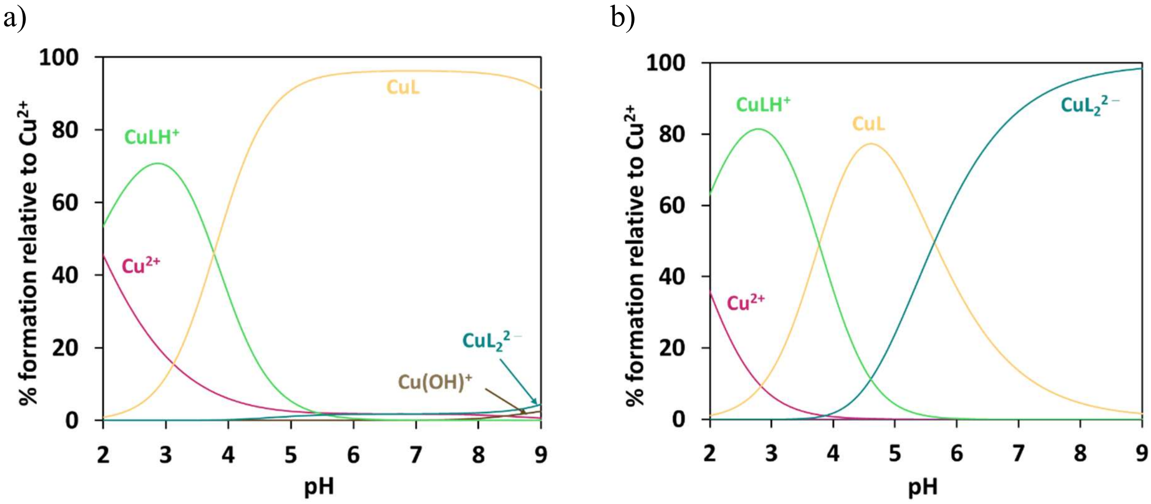

Figure S15. Distribution diagrams of $Cu_pL_qH_r$ species as a function of pH in the Cu^{2+}/8-HQA system in $KCl_{(aq)}$ at $I = 0.2$ mol dm^{-3} and $T = 298.2$ K. a) $c_L = c_{Cu} = 10^{-4}$ mol dm^{-3} and b) $c_L = 2 \times c_{Cu} = 10^{-4}$ mol dm^{-3}.

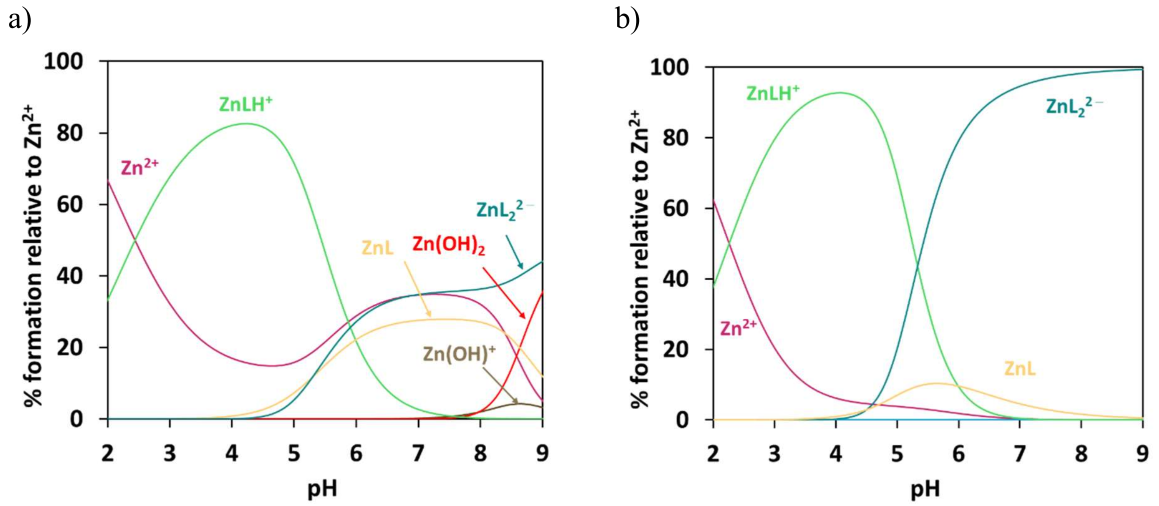

Figure S16. Distribution diagrams of $Zn_pL_qH_r$ species as a function of pH in the Zn^{2+}/8-HQA system in $KCl_{(aq)}$ at $I = 0.2$ mol dm^{-3} and $T = 298.2$ K. a) $c_L = c_{Zn} = 10^{-4}$ mol dm^{-3} and b) $c_L = 2 \times c_{Zn} = 10^{-4}$ mol dm^{-3}.

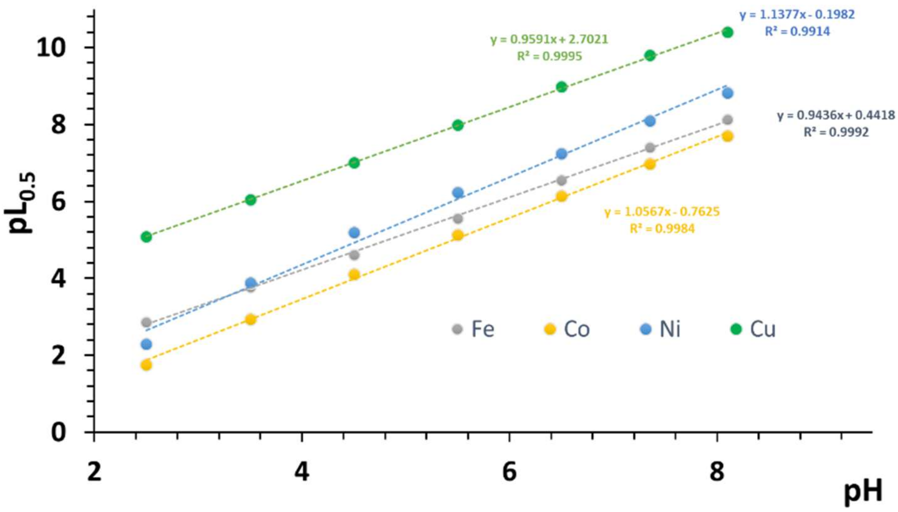

Figure S17. Sequestering ability ($pL_{0.5}$) of 8-HQA towards M^{2+} (M = Fe, Co, Ni, Cu), as a function of pH and corresponding linear trendlines, at $T = 298.2$ K and at $I = 0.2$ mol dm^{-3} in $KCl_{(aq)}$.

Table S4. $pL_{0.5}$ values for 8-HQA calculated at different pH values, at T = 298.2 K and at I = 0.2 mol dm^{-3} in $KCl_{(aq)}$.

**pH**	**$pL_{0.5}$**							
	**Fe^{3+} [a]**	**Ga^{3+} [b]**	**Mn^{2+}**	**Fe^{2+}**	**Co^{2+}**	**Ni^{2+}**	**Cu^{2+}**	**Zn^{2+}**
2.5	4.8	5.4	1.9	2.9	1.8	2.3	5.1	4.5
3.5	6.3	7.1	2.7	3.8	2.9	3.9	6.1	5.3
4.5	6.5	8.2	3.0	4.6	4.1	5.2	7.0	5.7
5.5	6.2	8.4	3.3	5.6	5.1	6.2	8.0	5.9
6.5	5.6	8.4	3.9	6.6	6.1	7.2	9.0	6.6
7.35	4.8	8.4	4.7	7.4	7.0	8.1	9.8	7.4
8.1	4.0	8.3	5.4	8.1	7.7	8.8	10.4	8.0
9.5	2.4	7.0	6.5	9.1	8.3	9.9	10.2	8.2

[a] From ref.[1]; [b] from ref.[6]

References

(1) Gama, S.; Frontauria, M.; Ueberschaar, N.; Brancato, G.; Milea, D.; Sammartano, S.; Plass, W. Thermodynamic study on 8-hydroxyquinoline-2-carboxylic acid as a chelating agent for iron found in the gut of Noctuid larvae. *New J. Chem.* **2018,** *42* (10), 8062-8073, DOI: 10.1039/c7nj04889k.

(2) Brown, P. L.; Ekberg, C. *Hydrolysis of metal ions*, Wiley-VCH Verlag GmbH & KGaA: Weinheim, 2016.

(3) Baes, C. F.; Mesmer, R. E. *The hydrolysis of cations*, John Wiley & Sons, Ltd: New York, 1976.

(4) Arena, K.; Brancato, G.; Cacciola, F.; Crea, F.; Cataldo, S.; De Stefano, C.; Gama, S.; Lando, G.; Milea, D.; Mondello, L.; Pettignano, A.; Plass, W.; Sammartano, S. 8-Hydroxyquinoline-2-Carboxylic Acid as Possible Molybdophore: A Multi-Technique Approach to Define Its Chemical Speciation, Coordination and Sequestering Ability in Aqueous Solution. *Biomolecules* **2020,** *10* (6), 930, DOI: 10.3390/biom10060930.

(5) Cukrowski, I. Polarographic Complex Formation Curves for Fully Inert Metal-Ligand System. *Electroanalysis* **1999,** *11* (9), 606-613.

(6) Ryza, I.; Granata, C.; Ribeiro, N.; Nalewajko-Sieliwoniuk, E.; Kiessling, A.; Hryniewicka, M.; Plass, W.; Godlewska-Zylkiewicz, B.; Cabo Verde, S.; Milea, D.; Gama, S. Ga complexes of 8-hydroxyquinoline-2-carboxylic acid: Chemical speciation and biological activity. *J Inorg Biochem* **2024,** *260*, 112670, DOI: 10.1016/j.jinorgbio.2024.112670.
